# Comprehensive Phytochemical Profiling and Chemotypic Variation Study of Three Medicinally Important Oncosiphon Species Indigenous to South Africa

**DOI:** 10.3390/plants15071047

**Published:** 2026-03-28

**Authors:** Tshwarelo R. Mathabatha, Maxleene Sandasi, Guy P. P. Kamatou, Weiyang Chen, Efficient Ncube, Bharathi Avula, Kumar Katragunta, Ikhlas A. Khan, Alvaro M. Viljoen

**Affiliations:** 1 Department of Pharmaceutical Sciences, Faculty of Science, Tshwane University of Technology, Private Bag X680, Pretoria 0001, South Africa; mathabathatr@tut.ac.za (T.R.M.); sandasim@tut.ac.za (M.S.); chenw@tut.ac.za (W.C.); ncubeen@tut.ac.za (E.N.); 2 SAMRC Herbal Drugs Research Unit, Tshwane University of Technology, Pretoria 0001, South Africa; 3 National Center for Natural Products Research, School of Pharmacy, University of Mississippi, University, MS 38677, USA; bavula@olemiss.edu (B.A.); kkatragu@olemiss.edu (K.K.); ikhan@olemiss.edu (I.A.K.); 4 Division of Pharmacognosy, Department of BioMolecular Sciences, School of Pharmacy, University of Mississippi, University, MS 38677, USA

**Keywords:** *Oncosiphon*, liquid chromatography–mass spectrometry, high–performance thin layer chromatography, 2D gas chromatography, chemometrics

## Abstract

The genus *Oncosiphon* (Asteraceae), consisting of aromatic herbs, is indigenous to southern Africa. *Oncosiphon* species have been documented in Khoi-San ethnobotany as herbal remedies for typhoid fever, pneumonia, and as diuretics. Research on the biological properties and comprehensive phytochemical profiling of these important *Oncosiphon* species is currently limited. This study was therefore undertaken to address the knowledge void in chemical profiling, through the application of various analytical techniques to analyse the volatile and non-volatile constituents of three South African *Oncosiphon* species. The aerial parts of *Oncosiphon suffruticosus* (n = 28), *O. grandiflorus* (n = 16), and *O. africanus* (n = 4) were collected from various locations in the Western Cape Province of South Africa. The stems and leaves (SL) were separated from the flowers (F) and analysed as distinct samples. The methanol: chloroform (1:1, *v*/*v*) extracts were prepared and analysed using ultra–high–performance liquid chromatography quadrupole time-of-flight time–of–flight mass spectrometry (UHPLC–QToF–MS) and a semi–automated high–performance thin–layer chromatography (HPTLC) system. Multivariate data analysis was performed on the UHPLC–QToF–MS data to determine interspecies chemical variation. Two-dimensional (2D) gas chromatography (GCxGC–ToF–MS) was used to determine the headspace volatile profiles of the intact aerial parts. The results show that the non-volatile profiles of the *Oncosiphon* species are characterised by amino acids, phenolic acids, flavonoids, sesquiterpene lactones, and fatty acid derivatives. The HPTLC profiles of *O. grandiflorus* and *O. africanus* are chemically more closely related, and *O. suffruticosus* has a distinct profile, which is supported by the chemometrics results of the flowers. The major headspace volatile compounds *in Oncosiphon* flowers are α-pinene, α-ocimene, eucalyptol, o-cymene, and artemisia alcohol, whereas the stems and leaves mainly consist of α-ocimene, eucalyptol, and yomogi alcohol.

## 1. Introduction

The Cape Floristic Region is the richest floral region in southern Africa, with approximately 4000 plant species used by the indigenous people of South Africa as herbal remedies [1]. Among these medicinal plants are shrubs of the genus *Oncosiphon* (Asteraceae), which harbours seven species in South Africa, namely, *O. sabulosus* (Wolley-Dod) Källersjö, *O. schlechteri* (Bolus ex Schltr.) Källersjö, *O. grandiflorus* (Thunb.) Källersjö, *O. inter-medius* (Hutch.) Källersjö, *O. piluliferus* (L.f.) Källersjö*, O. suffruticosus* (L.) Källersjö, and *O. africanus* (P.J.Bergius) Källersjö [2]. The term “*Oncosiphon*” combines two Greek words, “*oncos*” and “*siphon*”, meaning thick and tube, respectively. Collectively, the terms refer to the conspicuously swollen corolla tube that is characteristic of *Oncosiphon* species [3]. *Oncosiphon species* are generally referred to as “*stinkruid*” in Afrikaans because of their strong and unpleasant odour [4]. The plants grow up to 1 m in height, with flower heads that are 5 to 8 mm in diameter [5,6]. The feathery leaves and bright yellow flower heads make the plants conspicuous in any landscape. *Oncosiphon suffruticosus*, *O. piluliferus*, and *O. africanus* are popularly prepared as infusions and taken to treat ailments such as typhoid fever, rheumatic fever, influenza, asthma, and pneumonia [7]. The plants are also used as general tonics, anthelmintics, diuretics, diaphoretics, and applied topically as poultices for scorpion stings [5,7]. A few drops of fresh leaf juice are added to breast milk to relieve babies from colic and stomach cramps [6]. *Oncosiphon suffruticosus* was used as a remedy during the Spanish flu outbreak in 1918 [5].

Despite the medicinal significance of the three *Oncosiphon* species, detailed scientific research on their biological properties and chemical profiles remains limited. The chemistry of the indigenous *Oncosiphon* species has only been reported for *O. piluliferus* and *O. suffruticosus*. In a study by Pillay et al. [8], three germacrane lactones, namely, 4,5α–epoxy–6α–hydroxy–1(10)E,11(13)–germacradien–12,8α–olide, tatridin A (tavulin) and tanachin, and two eudesmanolides (desacetyl–β–cyclopyrethrosin and sivasinolide) were isolated from *O. piluliferus*. Adewinogo et al. [9] reported the major essential oil constituents of *O. suffruticosus* as camphor (31.2%), filifolone (14.0%), chrysanthenone (8.7%), eucalyptol (1,8–cineole) (7.9%), and terpinen–4–ol (7.4%), along with 11 other minor compounds constituting approximately 85% of the total composition. The above-mentioned reports confirm the scarcity of data on the chemistry of these medicinal plants, which is an important aspect in the quality control of herbal medicines, as it directly impacts the therapeutic outcomes as well as the safety profile of the plant.

Advanced chromatography techniques such as ultra-performance liquid chromatography coupled with mass spectrometry (UPLC–MS), high-performance thin layer chromatography (HPTLC), and gas chromatography with hyphenated mass spectrometry (GC–MS) enable precise separation, quantification, and identification of plant constituents. When combined with chemometrics data analysis, these techniques can reveal chemical variation between plant species [10]. In a study conducted by Acharya et al. [11], UPLC–MS in tandem with chemometrics analysis differentiated *Sutherlandia frutescens* from *S. microphylla* and predicted the chemical markers responsible for the observed variation. A similar approach was used in the quality assessment of several indigenous South African medicinal plants, distinguishing between closely related taxa such as *Pelargonium sidoides* and *P. reniforme* [12], three *Salvia* species [13], *Agathosma betulina* and *A. crenulata* [14], and *Harpagophytum procumbens* and *H. zeyheri* [15]. The current study, therefore, sought to contribute to knowledge on the three South African *Oncosiphon* species by documenting comprehensive phytochemical profiles of both the volatile and non-volatile constituents, using various chromatography techniques. In tandem with chemometrics analysis, the study highlights interspecies chemical variation and predicts chemical markers that distinguish the species.

## 2. Results

### 2.1. The HPTLC Fingerprint Profiles of Three Oncosiphon Species

#### 2.1.1. The HPTLC Chemical Profiles of the Flowers

**Figure 1:**
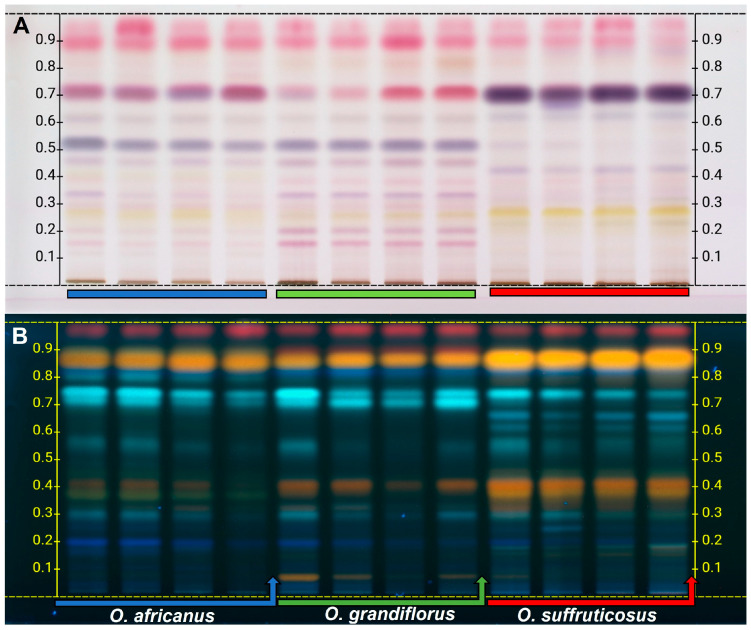
The HPTLC fingerprints of *Oncosiphon* flowers after derivatisation with (**A**) anisaldehyde and viewed under white light and (**B**) natural products reagent and viewed at 366 nm.

Typical HPTLC profiles of the three *Oncosiphon* species derivatised with solvent systems I and II and visualised with anisaldehyde reagent (A) and natural products (NP) reagent (B), respectively, are displayed in Figure 1. Chromatographic plate derivatised with anisaldehyde (Figure 1A) displayed bands in shades of pink, yellow, and violet/purple, which is consistent with the presence of flavonoid compounds, identified as marker constituents listed in Table 1. Wagner and Bladt [16] reported that flavonoids are detectable with anisaldehyde reagent, which produces distinctive colour reactions, typically yellow, pink, or violet/purple, following heating and observation under white light. A total of 11 bands were resolved on the HPTLC plate, and these occurred across the three *Oncosiphon* species (Figure 1A), with observable differences in the colour intensity. The majority of the compounds between Rf 0.10–0.80 occurred at low levels in *O. suffruticosus,* except for the compounds at Rf 0.28 (yellow) (kaempferide) and 0.70 (purple) (unknown), which occurred in higher amounts in this species. A distinct marker compound for *O. suffruticosus* was observed at Rf 0.42 (light purple), which was identified as eupalitin. *Oncosiphon grandiflorus* and *O. africanus* showed similar profiles, with the majority of the compounds occurring at higher levels in *O. grandiflorus*. Some of the compounds shared between the two species include 5,6,4′–trihydroxy–7,8,3′–trimethoxyflavone (Rf 0.16), tetrahydroxy–methoxyflavone (methyl quercetin) (Rf 0.20), and tricin–glucuronoside (Rf 0.35), while others remain unknown. A marker compound (unknown) for *O. africanus* was observed at Rf 0.43 (yellow), while *O. grandiflorus* did not show any distinct bands from the other two species (Figure 1A, Table 1).

Representative HPTLC profiles developed using solvent system II and derivatised with NP reagent are presented in Figure 1B. The plate shows yellow, red, and blue fluorescent bands, which indicate the presence of phenolic compounds and flavonoids. Wagner and Bladt [16] reported that phenolic compounds and flavonoids commonly display characteristic blue, yellow, and red fluorescence under 366 nm, following derivatisation with the NP reagent. Eleven common bands (compounds) at varying concentrations are shared among the three species (Rf 0.22, 0.30, 0.32, 0.37, 0.42, 0.58, 0.70, 0.72, 0.82, 0.85, and 0.97) (Figure 1B, Table 1). The profile for *O. suffruticosus* is more distinct, with characteristic bands of unknown compounds at Rf 0.19 (yellow), 0.62 (light blue), and 0.68 (light blue), which were absent in the other two species. *Oncosiphon grandiflorus* and *O. africanus* displayed unique bands at Rf 0.90 (red) and 0.80 (light blue), respectively. The results therefore demonstrate that the flowers of *O. suffruticosus* have a distinct profile, consisting of higher levels of phenolics and flavonoids. However, the chromatographic pattern within each species is generally conserved between samples with some quantitative variation.

#### 2.1.2. The HPTLC Chemical Profiles of the Stems and Leaves

**Figure 2:**
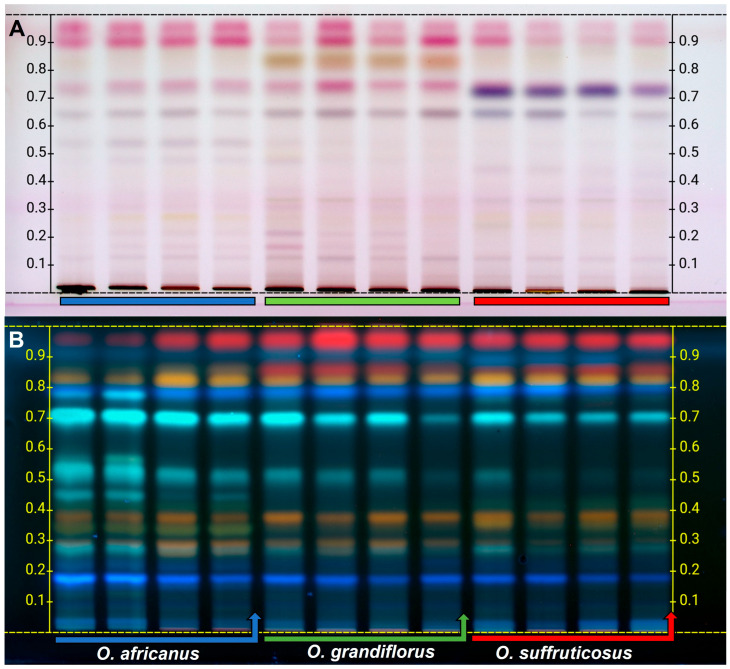
The HPTLC fingerprints of *Oncosiphon* stems and leaves after derivatisation with (**A**) anisaldehyde and viewed under white light and (**B**) natural products reagent and viewed at 366 nm.

Under white light, the stems and leaves exhibited quantitative differences across the three species within the Rf range from 0.60 to 0.95 (Figure 2A). Kaempferide (Rf 0.28) was present in all the species, while distinct, unidentified bands were observed at Rf 0.45 (light purple) in *O. suffruticosus* and at Rf 0.84 (yellow) in *O. grandiflorus* (Table 2). Generally, the stems and leaves produced low-intensity bands following derivatisation with anisaldehyde, indicating that the flavonoid composition is lower compared to the flowers. After derivatisation of the stems and leaves with NP reagent and visualisation under 366 nm UV light (Figure 2B), the HPTLC plate revealed common bands of varying intensities between Rf 0.19–0.39 and 0.70–0.95, as listed in Table 2. The qualitative differences were observed at Rf 0.37 (light yellow) and Rf 0.42 (light yellow) for *O. africanus* and *O. suffruticosus*, at Rf 0.48 (light blue) and Rf 0.56 (light blue) for *O. africanus*, and a red band that appeared only in *O. grandiflorus* and *O. suffruticosus* at Rf 0.86. The majority of these compounds were observed in *O. africanus*, which displayed the highest number of bands. Dicaffeoylquinic acid (Rf = 0.72), as well as several flavonoids detected at Rf 0.35, including dihydroxy–dimethoxyflavone, an isomer of isorhamnetin, syringetin or spinacetin, and kaempferide, were common to all three species. Like the HPTLC profiles of the flowers, the stems and leaves of all three species showed minimal intraspecies variation.

### 2.2. LC–DAD-QToF–MS Characterisation and Chemotaxonomic Differentiation of Oncosiphon Species

Structural assignments were accomplished through a comprehensive interpretation of high-resolution LC–QToF–MS data acquired in both positive and negative ionisation modes (Table 3). Accurate mass measurements were obtained with mass errors generally within ±5 ppm, providing high confidence in elemental composition determination. Experimental *m*/*z* values were systematically compared with theoretical exact masses, and the calculated values are presented in parentheses in Table 3 to demonstrate the close agreement between observed and predicted ions. In addition to accurate mass confirmation, compound identification relied on characteristic MS/MS fragmentation pathways, including diagnostic neutral losses (e.g., H_2_O, CO_2_, CO, CH_3_, and sugar moieties) and structurally informative product ions. These fragmentation patterns were interpreted in accordance with previously reported literature data for related compounds. Whenever authentic standards were available, chromatographic retention behaviour and MS/MS spectra were directly compared to confirm identity.

**Figure 3:**
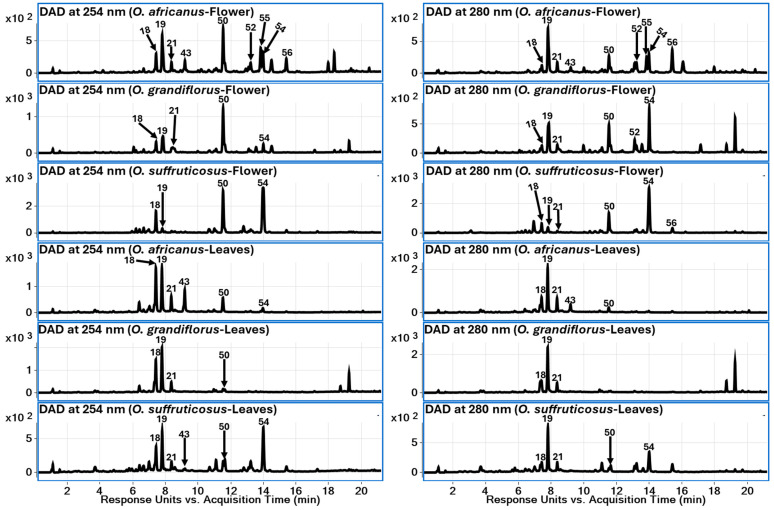
LC-DAD chromatograms of *Oncosiphon* species (flower and leaf extracts) at 254 nm and 280 nm (high-intensity peaks were labelled).

LC–QToF–MS profiling enabled the putative identification of 83 metabolites in flower and leaf extracts of *O. africanus*, *O. grandiflorus*, and *O. suffruticosus*. These metabolites were classified into five major chemical groups: amino acids (**1**–**10**), phenolic acids (**11**–**22**), flavonoids (**23**–**56**), sesquiterpene lactones (**57**–**74**), and fatty acid derivatives (**75**–**83**). The detected compounds covered a wide polarity range, reflecting the metabolic diversity of the species investigated. The LC-DAD chromatographic profiles (comparative) of three *Oncosiphon* species (flower and leaf) at 254 nm and 280 nm are provided in Figure 3.

#### 2.2.1. Amino Acids (Compounds **1**–**10**)

Amino acids constituted the earliest eluting metabolites in the chromatographic profile (RT 1.17–2.87 min), consistent with their high polarity and low hydrophobicity. All amino acids were detected predominantly in positive ionisation mode as protonated molecular ions [M+H]^+^, whereas no significant signals were observed in negative mode under the applied conditions. Accurate mass measurements showed excellent agreement between experimental and theoretical values (≤5 ppm), supporting confident elemental composition assignment.

Serine (**1**) displayed a protonated molecular ion at *m*/*z* 106.0507 and characteristic dehydration fragments at *m*/*z* 88.0392 [M+H-H_2_O]^+^ and 70.0289 [M+H-2H_2_O]^+^, reflecting the presence of a hydroxyl group prone to water loss. Threonine (**7**), another hydroxylated amino acid, showed similar dehydration behaviour with a base fragment at *m*/*z* 102.0541 [M+H-H_2_O]^+^, followed by further loss of CO to yield *m*/*z* 74.0603. Proline (**2**) exhibited *m*/*z* 116.0701 with a dominant fragment at *m*/*z* 70.0659, corresponding to sequential loss of H_2_O and CO. This fragmentation is typical of cyclic imino acids, where ring-cleavage and decarboxylation generate stable product ions. Valine (**3**) and isoleucine/leucine (**5**–**6**) showed protonated molecular ions at *m*/*z* 118.0857 and 132.1023, respectively, with characteristic neutral losses of H_2_O and CO to yield fragments at *m*/*z* 72.0818 and 86.0961. These fragmentation pathways are consistent with α-amino acid decarboxylation and side-chain cleavage mechanisms commonly reported for branched-chain amino acids [17].

Aromatic amino acids exhibited more diagnostic fragmentation patterns due to the stability of aromatic cations. Tyrosine (**4**) generated *m*/*z* 182.0810 and prominent fragments at *m*/*z* 165.0541 [M+H-NH_3_]^+^ and 147.0433 [M+H–NH_3_-H_2_O]^+^, indicating sequential loss of ammonia and water. Additional fragments at *m*/*z* 136.0752 and 123.0435 further supported cleavage adjacent to the phenolic ring. Phenylalanine (**9**) showed a protonated ion at *m*/*z* 166.0865 and produced a characteristic benzyl-type fragment at *m*/*z* 91.0547, along with *m*/*z* 120.0824 arising from combined loss of H_2_O and CO. The formation of *m*/*z* 91.0547 (C_7_H_7_^+^) is particularly indicative of a substituted phenyl group. Tryptophan (**10**), containing an indole moiety, displayed *m*/*z* 205.0971 (calcd *m*/*z* 205.0972) and sequential fragmentation involving loss of NH_3_ (*m*/*z* 188.0702) and CH_2_CO (*m*/*z* 146.0589), consistent with cleavage adjacent to the indole side chain. The presence of fragments at *m*/*z* 118.0638 and 91.0527 further confirmed indole ring-related fragmentation pathways [17].

Pipecolic acid (**8**), a non-proteinogenic cyclic amino acid, showed *m*/*z* 130.0869 [M+H]^+^ and characteristic dehydration and decarbonylation fragments at *m*/*z* 112.0761 and 84.0814, consistent with ring cleavage [18].

From a distribution perspective, all identified amino acids were detected in both flower and leaf extracts of *O. africanus*, *O. grandiflorus*, and *O. suffruticosus*. Their ubiquitous occurrence across species and tissues indicates that these metabolites represent conserved primary metabolic components rather than chemotaxonomic markers.

#### 2.2.2. Phenolic Acids (Compounds **11**–**22**)

Phenolic acids constituted a prominent group of secondary metabolites in the investigated species, mainly represented by hydroxycinnamic acid derivatives and quinic acid conjugates. These compounds were preferentially detected in negative ionisation mode as deprotonated molecular ions [M-H]^−^, which provided enhanced sensitivity and structurally informative fragmentation patterns typical of phenolic acids.

Quinic acid (**11**) exhibited a characteristic [M-H]^−^ ion at *m*/*z* 191.0557 and diagnostic fragments at *m*/*z* 173.0459 [M-H-H_2_O]^−^, 111.0468, and 93.0352, consistent with sequential dehydration and ring-cleavage pathways commonly reported for cyclitol structures [19]. Chlorogenic acid isomers (**12**–**14**), corresponding to caffeoylquinic acids (C_16_H_18_O_9_), showed [M-H]^−^ at *m*/*z* 353.0873 and produced prominent fragment ions at *m*/*z* 191.0558 (quinic acid moiety) and 179.0348 (caffeic acid moiety) [20,21]. Additional fragments at *m*/*z* 161.0246 and 135.0452 arose from dehydration and decarboxylation of the caffeoyl unit. The relative intensities of *m*/*z* 191 and 179 are diagnostic for distinguishing positional isomers of chlorogenic acids (3-*O*-, 4-*O*-, and 5-*O*-caffeoylquinic acids), as described in established LC–MS fragmentation rules for hydroxycinnamoylquinic acids [20,21]. The presence of the sodium adduct [M+Na]^+^ at *m*/*z* 377.0844 in positive mode further supported structural assignment.

Di-caffeoylquinic acid (DCQA) isomers (**18**–**21**) displayed [M-H]^−^ at *m*/*z* 515.1191 and characteristic product ions at *m*/*z* 353.0876 (loss of one caffeoyl residue), 191.0559 (quinic acid), and 179.0347 (caffeic acid), confirming the presence of two caffeoyl substituents [20,21]. Coumaroylquinic acid (**16**) showed a deprotonated ion at *m*/*z* 337.0929 with diagnostic fragments at *m*/*z* 191.0555 (quinic acid), 173.0461 [quinic acid-H_2_O]^−^, and 163.0412 (coumaric acid anion) [20,21]. Similarly, feruloylquinic acid (**17**) produced [M-H]^−^ at *m*/*z* 367.1039 and characteristic fragment ions at *m*/*z* 193.0531 (ferulic acid), 191.0559 (quinic acid), and 149.0581 [ferulic acid-CO_2_]^−^. The presence of *m*/*z* 134.0362 further supported methoxy-substituted hydroxycinnamic acid fragmentation, which typically involves demethylation and decarboxylation pathways [20,21].

Free hydroxycinnamic acids were also identified. Caffeic acid (**15**) exhibited [M-H]^−^ at *m*/*z* 179.0347 and a prominent fragment at *m*/*z* 135.0450 corresponding to decarboxylation (−44 Da), a well-known fragmentation pathway for cinnamic acid derivatives. Ferulic acid (**22**) showed [M-H]^−^ at *m*/*z* 193.0510 and diagnostic fragments at *m*/*z* 178.0261 (loss of CH_3_), 149.0611 (loss of CO_2_), and 134.0379, consistent with methoxy-substituted cinnamic acid fragmentation mechanisms reported in the literature [19].

From a distribution perspective, phenolic acids were detected across flower and leaf tissues of *O. africanus*, *O. grandiflorus*, and *O. suffruticosus*. Mono- and di-caffeoylquinic acids were widely distributed and represented major phenolic constituents. However, certain compounds, such as ferulic acid and feruloylquinic acid derivatives, were more prevalent in leaf extracts, suggesting tissue-dependent biosynthetic regulation. Although phenolic acids were not exclusively species-specific, subtle qualitative and relative abundance differences contributed to the overall metabolic fingerprint of each species.

Overall, the phenolic acid profile was characterised by a predominance of hydroxycinnamoylquinic acid derivatives, whose fragmentation patterns closely matched established LC–MS diagnostic rules for this class. The combination of accurate mass data and structurally informative MS/MS spectra enabled reliable annotation and supported their role as key phenolic constituents in *Oncosiphon* species.

#### 2.2.3. Flavonoids (Compounds **23**–**56**)

Flavonoids constituted one of the most structurally diverse and chemotaxonomically informative classes detected in the investigated taxa, comprising flavan-3-ols, flavonols, flavones, methoxylated derivatives, and a wide array of glycosylated conjugates. These compounds were detected in both ionisation modes; however, the negative ion mode generally yielded more abundant, structurally diagnostic product ions for flavonol and flavone derivatives. Structural assignments were supported by accurate mass measurements (≤5 ppm error), characteristic neutral losses of sugar moieties, and well-established Retro-Diels–Alder (RDA) fragmentation pathways of the flavonoid C-ring [20,21,22,23,24,25,26,27].

##### Flavan-3-ols

Gallocatechin/epigallocatechin (**23**–**24**) type flavan-3-ol was identified based on the deprotonated molecular ion at *m*/*z* 305.0671. Their MS/MS spectra exhibited characteristic fragment ions at *m*/*z* 221.0462, 179.0371, 137.0249, and 125.0221, which arise from RDA cleavage and heterocyclic ring fission of the flavan-3-ol skeleton [22]. These fragmentation pathways are consistent with previously reported LC–MS/MS behaviour of catechin-type compounds [22]. The presence of diagnostic A-ring fragments further confirmed the trihydroxylated B-ring substitution pattern.

##### Flavonol Glycosides

Flavonol glycosides were prominently represented by quercetin, kaempferol, and isorhamnetin derivatives. Quercetin–3–*O*–rutinoside (rutin, **25**) exhibited a deprotonated molecular ion at *m*/*z* 609.1455. MS/MS analysis showed sequential neutral losses of rhamnose (−146 Da) and glucose (−162 Da), yielding the quercetin aglycone ion at *m*/*z* 301.0345. In positive mode, the protonated molecular ion at *m*/*z* 611.1604 generated fragments at *m*/*z* 465.1025 [M+H-Rha]^+^ and 303.0499, confirming the rutinoside substitution. Such stepwise sugar losses are well established for *O*-glycosylated flavonols [20].

Monohexoside derivatives of quercetin (**27**) showed [M-H]^−^ at *m*/*z* 463.0881 and produced a dominant fragment at *m*/*z* 301.0351 corresponding to the aglycone, confirming hexose conjugation (−162 Da) [20]. Similarly, isorhamnetin 3–*O*–hexosides (**40, 41**) exhibited [M-H]^−^ at *m*/*z* 477.1035 and yielded *m*/*z* 315.0502 (isorhamnetin aglycone), along with *m*/*z* 300.0248 reflecting loss of a methyl group (−15 Da). The additional fragments at *m*/*z* 151 and 107 arose from RDA cleavage of the aglycone and are characteristic of flavonol structures [21].

Isorhamnetin dihexoside isomers (**30**–**31**) displayed [M-H]^−^ at *m*/z 639.1563 and sequential sugar losses, generating *m*/*z* 477.1050 and 315.0507, confirming the presence of two hexose units. The fragmentation patterns were consistent with previously reported behaviour of di-*O*-glycosylated flavonols [21].

##### Flavone Glycosides and Glucuronides

Flavone glycosides, including luteolin and kaempferol derivatives, were widely distributed. Luteolin/kaempferol–*O*–rutinoside (**32**–**33**) produced [M-H]^−^ at *m*/*z* 593.1509 and fragments at *m*/*z* 447.0941 [M-H-Rha]^−^ and 285.0395 (aglycone), confirming disaccharide substitution [21]. Monohexosides (**37, 38**) exhibited [M-H]^−^ at *m*/*z* 447.0929 and a diagnostic aglycone ion at *m*/*z* 285.0401. These fragmentation pathways correspond to typical *O*-glycosidic bond cleavage [20]. Luteolin glucuronide (**39**) showed [M-H]^−^ at *m*/*z* 461.0723 and produced *m*/*z* 285.0403 following loss of glucuronic acid (−176 Da), a neutral loss widely recognised as indicative of glucuronidation [21,24,25]. Tricin–glucuronoside (**43**) generated *m*/*z* 329.0666 (aglycone) and *m*/*z* 314.0426 [aglycone–CH_3_]^−^, supporting both methylation and glucuronide substitution [25].

##### Aglycones and Methoxylated Flavonoids

Aglycone flavonoids such as quercetin (**46**), luteolin (**47**), kaempferol (**48**), taxifolin (**34**), eriodictyol (**45**), and apigenin (**51**) were identified based on characteristic [M-H]^−^ ions and RDA fragmentation patterns. Quercetin (**46**) exhibited [M-H]^−^ at *m*/*z* 301.0362 and diagnostic RDA fragments at *m*/*z* 179.0350 and 151.0039. Luteolin/kaempferol (**47, 48**) showed *m*/*z* 285.0401 with fragments at *m*/*z* 151.0035 and 133.0290, consistent with A- and B-ring cleavages. Apigenin (**51**) produced [M-H]^−^ at *m*/*z* 269.0455 and fragments at *m*/*z* 151.0037 and 117.0343, typical of flavone fragmentation. Compounds **45**–**48** and **51** were identified using reference standards.

Methoxylated flavones, including eupalitin (**52**–**53**) and trimethoxyflavone derivatives (**55**), were characterised by sequential losses of methyl radicals (–15 Da) and demethylated fragments, a well-documented behaviour for *O*-methylated flavonoids [27]. For example, eupalitin exhibited [M-H]^−^ at *m*/*z* 329.0666 and fragments at *m*/*z* 314.0420 [M-H-CH_3_]^−^ and 299.0192 [M-H-2CH_3_]^−^, along with RDA fragments at *m*/*z* 151.0036. These patterns clearly supported methoxy substitution on the flavone backbone [27].

Flavonoids were detected in both flower and leaf extracts of *O. africanus*, *O. grandiflorus*, *and O. suffruticosus*. While many compounds were shared among species, qualitative differences in glycosylation type (rutinoside, hexoside, glucuronide) and degree of methoxylation were observed. Flower tissues generally exhibited greater diversity in methoxylated flavones and complex glycosides, whereas leaves showed comparatively simpler glycosylation patterns.

Overall, flavonoid identification was strongly supported by accurate mass measurements, diagnostic neutral losses of sugar moieties, and characteristic RDA fragmentation pathways. Although flavonoids contributed to tissue-level and interspecific metabolic variation, their chemotaxonomic significance was secondary to that of sesquiterpene lactones, which exhibited more pronounced qualitative differences among the three *Oncosiphon* species.

#### 2.2.4. Sesquiterpene Lactones (Compounds **57**–**74**)

The *Oncosiphon* species analysed (*O. africanus*, *O. grandiflorus*, and *O. suffruticosus*) contain a chemically diverse array of sesquiterpene lactones (SL’s), predominantly of the germacranolide and guaianolide types, as identified by LC-QToF-MS. These compounds are characterised by neutral losses of water (H_2_O), carbon dioxide (CO_2_), carbon monoxide (CO), and acetate (Ac) groups, which are diagnostic in positive ionisation mode [28,29,30,31].

Compounds **57**–**62** (C_15_H_20_O_4_), assigned as hydroxylated germacranolide isomers, exhibited a protonated ion at *m*/*z* 265.1431 and an ammonium adduct at *m*/*z* 282.1700. Their fragmentation pattern showed successive dehydration to *m*/*z* 247.1332 [M+H-H_2_O]^+^ and *m*/*z* 229.1225 [M+H-2H_2_O]^+^, followed by decarboxylation to *m*/*z* 221.1535 [M+H-CO_2_]^+^ and combined losses to *m*/*z* 203.1433 [M+H-H_2_O-CO_2_]^+^. Characteristic low-mass fragments at *m*/*z* 149.0965 (C_10_H_13_O^+^) and 121.1014 (C_9_H_13_^+^) supported cleavage of the germacrane skeleton [28,29,30,31]. These metabolites were consistently detected in flowers and leaves of *O. africanus* and *O. grandiflorus*, indicating close chemotaxonomic similarity between these two species. In negative mode, the deprotonated precursor at *m*/*z* 263.1289 produced a major fragment at *m*/*z* 219.1387 corresponding to the loss of CO_2_ [M-H-CO_2_]^−^. Compound **63** (C_15_H_18_O_4_), tentatively identified as isoepoxyestafiatin or an isomer, showed [M+H]^+^ at *m*/*z* 263.1280 and [M+Na]^+^ at *m*/*z* 285.1101. MS/MS fragmentation included dehydration to *m*/*z* 245.1179 and decarboxylation to *m*/*z* 219.1388, followed by further loss to *m*/*z* 201.1272 [28,29,30,31]. The compound was observed in both organs of *O. africanus* and *O. grandiflorus* and in trace amounts in *O. suffruticosus*. Compound **65** (C_15_H_20_O_5_), assigned as 4,5-epoxy-8,13-dihydroxy-1(10),7(11)-germacradien-12,6-olide, exhibited a protonated molecular ion at *m*/*z* 281.1358. Its MS/MS spectrum was dominated by sequential water losses to *m*/*z* 263.1277 and 245.1142, followed by decarboxylation to *m*/*z* 237.1501 and combined dehydration/decarboxylation to *m*/*z* 201.1271 [28,29,30,31]. This metabolite was characteristic of *O. grandiflorus*. The eudesmanolide derivative santamarin or reynosin (Compound **66**, C_15_H_20_O_3_) showed [M+H]^+^ at *m*/*z* 249.1488 and [M+Na]^+^ at *m*/*z* 271.1305. Fragmentation involved dehydration to *m*/*z* 231.1382 and decarboxylation to *m*/*z* 205.1589, typical of eudesmanolide frameworks [28,29]. This compound was primarily detected in *O. suffruticosus*. Dehydrocostunolide or germacranolide derivative (Compound **68/69**, C_15_H_18_O_2_) displayed [M+H]^+^ at *m*/*z* 231.1381 with dehydration to *m*/*z* 213.1276 and further fragmentation to *m*/*z* 187.1481 [28,29,30]. Compound **70** (C_15_H_16_O_3_), representing guaianolide derivatives, showed [M+H]^+^ at *m*/*z* 245.1171 and characteristic fragments at *m*/*z* 227.1071 [M+H-H_2_O]^+^ and 201.1277 [M+H-CO_2_]^+^ [28,29,30,31]. Matricarin (Compound **72**, C_17_H_20_O_5_) exhibited [M+H]^+^ at *m*/*z* 305.1386 and fragmentation consistent with acetyl cleavage and lactone rearrangements [31]. These guaianolides were predominantly associated with *O. suffruticosus*, suggesting species-level differentiation. A saturated analog, 1α,6α-dihydroxy-4E,10(14)-germacradien-12,8α-olide (**73**), was also identified at a deprotonated mass of *m*/*z* 265.1451 [28,29,30,31]. Its fragmentation pattern confirmed the expected losses of water and CO_2_, producing fragments at *m*/*z* 247.1344 and 221.1547, consistent with hydroxylated germacranolide structures. Costunolide or germacranolide derivative (Compound **74**, C_15_H_20_O_2_) was observed with [M+H]^+^ at *m*/*z* 233.1538. Diagnostic ions at *m*/*z* 215.1432 [M+H-H_2_O]^+^ and 189.1642 [M+H-CO_2_]^+^, along with hydrocarbon fragments at *m*/*z* 145.1013 and 131.0856, confirmed the germacranolide structure [28,30]. Costunolide (or germacranolide derivative) was high in *O. suffruticosus* and present in trace amounts in the other species, supporting its potential as a chemotaxonomic marker.

Overall, sesquiterpene lactones exhibited species-dependent distribution, with *O. africanus* and *O. grandiflorus* enriched in hydroxylated germacranolides, whereas *O. suffruticosus* showed greater abundance of guaianolides and costunolide-type derivatives.

#### 2.2.5. Fatty Acid Derivatives (Compounds **75**–**83**)

Fatty acid derivatives were primarily detected in negative ion mode as deprotonated molecules [M-H]^−^. Trihydroxy-octadecadienoic acid (Compound **75**, C_18_H_32_O_5_) showed [M-H]^−^ at *m*/*z* 327.2177, undergoing sequential dehydration to *m*/*z* 309.2070, 291.1977, and 273.1839 [32]. The saturated analogue (Compound **76**, C_18_H_34_O_5_) exhibited [M-H]^−^ at *m*/*z* 329.2323 with similar fragmentation behaviour [32]. These trihydroxy fatty acids were broadly distributed across all species and organs. Hydroxy-octadecadienoic acid (Compounds **77**–**79,** C_18_H_32_O_3_) presented [M-H]^−^ at *m*/*z* 295.2278 and fragments at *m*/*z* 277.2171 [M-H-H_2_O]^−^, 251.2361 [M-H-CO_2_]^−^, and 233.2275, consistent with hydroxyl group rearrangements and neutral losses along the C-18 chain [33]. The oxo-octadecadienoic acid isomers (Compounds **81**–**83**, C_18_H_30_O_3_) showed [M-H]^−^ at *m*/*z* 293.2122 with diagnostic losses to *m*/*z* 275.2016, 249.2199, 205.1594, and 167.1063, reflecting the oxo substitution and unsaturation within the fatty acid chain [33]. The dicarboxylic fatty acid 9-octadecenedioic acid (Compound **80**, C_18_H_32_O_4_) exhibited [M-H]^−^ at *m*/*z* 311.2224 and decarboxylation to *m*/*z* 267.2330 [34]. Overall, these fatty acid derivatives were consistently found across leaves and flowersof all three *Oncosiphon* species.

#### 2.2.6. Chemotaxonomic Implications

While amino acids and fatty acid derivatives were ubiquitously distributed and largely conserved, interspecific differentiation among the three *Oncosiphon* species was primarily driven by variations in sesquiterpene lactone composition and, to a lesser extent, flavonoid substitution patterns. Flowers exhibited particularly distinct sesquiterpene lactone profiles, highlighting tissue-specific metabolic specialisation. The integrated evaluation of accurate mass data and diagnostic MS/MS fragmentation patterns provided robust structural annotation and revealed clear metabolic signatures that support chemotaxonomic discrimination within the genus *Oncosiphon*.

### 2.3. Untargeted Chemometrics Analysis of the UPLC–MS Data

#### 2.3.1. Interspecies Chemical Variation Based on Flowers

**Figure 4:**
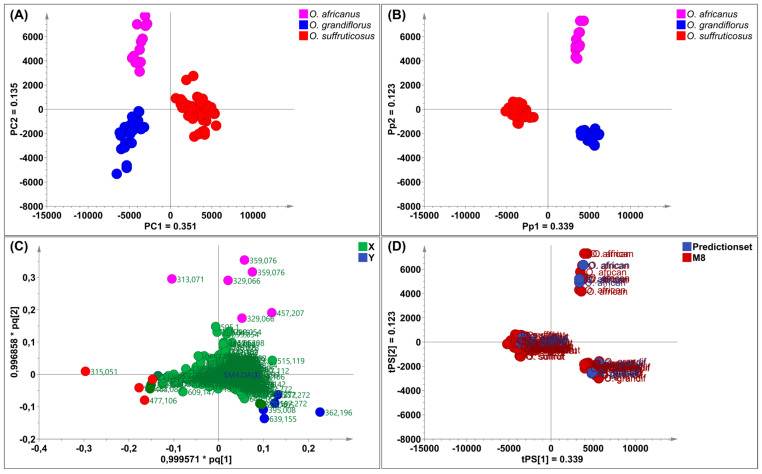
(**A**) PCA scores scatter plot of *Oncosiphon* flowers (**B**) an OPLS–DA scores plot indicating three separate clusters corresponding to the three *Oncosiphon* species, (**C**) the loadings plot derived from OPLS–DA analysis, indicating variables (marker compounds) corresponding to each species and (**D**) a prediction scores scatter plot showing correct identification of validation samples.

**Figure 5:**
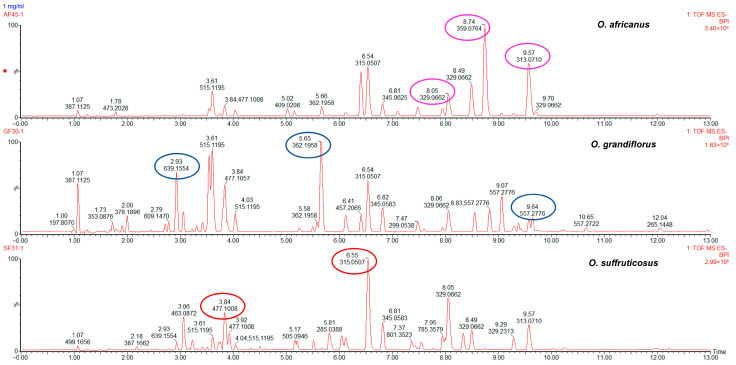
Representative UPLC–MS chromatograms of *Oncosiphon* flowers indicating the presence of chemical markers that distinguish the three species: *O. africanus* (pink), *O. grandiflorus* (blue), and *O. suffruticosus* (red) as revealed in the OPLS–DA loadings plot.

Principal component analysis was applied to reveal chemical variation between the *Oncosiphon* flowers. The UPLC–MS data matrix comprised 88 observations (N) and 3174 X–variables. The PCA model, characterised by six principal components, explained 70.3% of the total variation in the X–matrix (R^2^_cum_ 0.703) and demonstrated a 59.6% predictive ability (Q^2^_cum_ 0.596). The scores scatter plot of PC1 vs. PC2 (Figure 4A) revealed three distinct clusters that correspond to the three species. Samples of *O. suffruticosus* (red) clustered on the positive PC1, while *O. africanus* (pink) and *O. grandiflorus* (blue) clustered distinctly on the negative PC1. The chemical variation modelled along PC1 was 35.1%, while *O. africanus* and *O. grandiflorus*, separated along PC2, had a chemical variation of 13.5%. Following PCA modelling, a supervised OPLS–DA model was constructed. The scores plot (Figure 4B), coloured according to the three classes (species), indicates that *O. africanus* and *O. grandiflorus* separated and occupied positive PC1, while *O. suffruticosus* occupied a quadrant in negative PC1 (Figure 4B). The variation along the first predictive component (Pp1) was 33.9%, and further separation between the *O. africanus* and *O. grandiflorus* samples along Pp2 was modelled by 12.3%. The OPLS–DA model displayed a good predictive ability of 96.8% (Q^2^_cum_ 0.968). The clustering pattern confirms the HPTLC findings that the chemistry of O. *suffruticosus* flowers is more distinct compared to the other two species. The corresponding loadings plot revealed variables (marker compounds) responsible for the observed differences between the three species (Figure 4C). Variables located on the extreme ends of the loadings plot and colour-coded pink (*O. africanus*), blue (*O. grandiflorus*), and red (*O. suffruticosus*) are associated with their respective species, occupying the corresponding position in the OPLS-DA scored plot (Figure 4B). External validation of the model using test samples (blue) confirmed model reliability in accurately predicting the identities of the test samples (Figure 4D). The marker compounds identified on the loadings plot were identified through retention time/mass-to-charge ratio pairs (Rt/*m*/*z*) and are listed in Table 4. For *O. africanus*, three compounds were indicated as markers, and tentatively identified as eupalitin, 5,6,4′–trihydroxy–7,8,3′–trimethoxyflavone, and dihydroxy–dimethoxyflavone. Three marker compounds were indicated for *O. grandiflorus*, namely rhamnetin sophoroside and two other unidentified compounds. For *O. suffruticosus*, isorhamnetin 3–*O*–glucoside and methyl quercetin were revealed as distinguishing markers. Some of these markers correspond to the results obtained in the HPTLC analysis; the sensitivity of UHPLC–MS enabled the identification of more compounds. The UHPLC–MS profiles of the flower samples confirmed that the marker compounds vary quantitatively, as displayed in Figure 5.

#### 2.3.2. Interspecies Chemical Variation Based on Stems and Leaves

**Figure 6:**
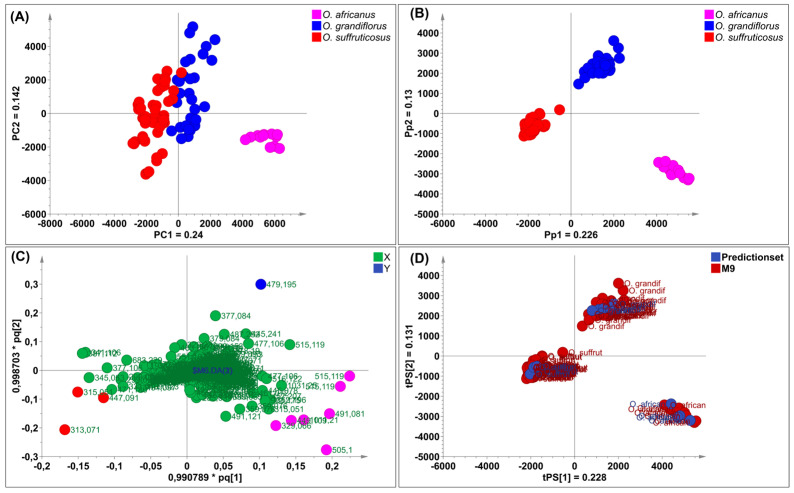
(**A**) PCA scores scatter plot of *Oncosiphon* stems and leaves (**B**) an OPLS–DA scores plot indicating three separate clusters corresponding to the three *Oncosiphon* species, (**C**) the loadings plot derived from OPLS–DA analysis, indicating variables (marker compounds) corresponding to each species and (**D**) a prediction scores scatter plot showing correct identification of validation samples.

**Figure 7:**
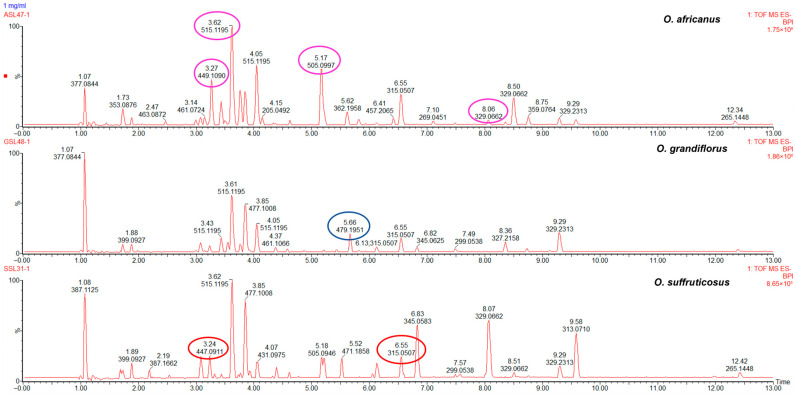
Representative UPLC-MS chromatograms of *Oncosiphon* stems and leaves indicating the presence of chemical markers that distinguish the three species: *O. africanus* (pink), *O. grandiflorus* (blue), and *O. suffruticosus* (red) as revealed in the OPLS-DA loadings plot.

The PCA model built on a data matrix comprising 98 observations (N) and 3174 X–variables, was characterised by seven principal components, which explained 71.6% of the total variation in the X–matrix (R^2^_cum_ 0.716) and 57.7% predictive ability (Q^2^_cum_ 0.577). The largest variation modelled along PC1 (24.0%) separated *O. africanus* from the other two species, while 14.2% variation was observed along PC2 (Figure 6A). The observed clustering pattern suggests that the stems and leaves profiles of *O. grandiflorus* and *O. suffruticosus* are closely related. Applying the supervised OPLS–DA, the model revealed a cumulative 52.3% variation in X (R^2^X_cum_ = 0.523), 95.7% (R^2^Y_cum_ = 0.957) in Y, and a good predictive ability of 96.7% (Q^2^_cum_ = 0.967). The 22.6% variation modelled along Pp1 was responsible for chemical differences between *O. africanus* and the other two species, while 13% of the variation in the chemical data was explained by Pp2, which separated *O. grandiflorus* and *O. suffruticosus* (Figure 6B). The corresponding loadings plot (Figure 6C) constructed from the OPLS–DA model revealed distinguishing markers for each of the three species. The Rt/*m*/*z* pairs and tentative identities of these markers are listed in Table 5. External validation showed that the OPLS–DA model accurately predicted the test samples into the correct species cluster (Figure 6D). The chemical markers for *O. africanus* were tentatively identified as dicaffeoylquinic acid, tricin–glucuronoside, eupalitin, and two unknown compounds. In *O. grandiflorus*, one unknown distinguishing marker was revealed, while three markers for *O. suffruticosus* were tentatively identified as kaempferol glucoside, methyl quercetin, and dihydroxy–dimethoxyflavone. These distinguishing chemical markers confirmed that the three species differ predominantly quantitatively rather than qualitatively, with *O. suffruticosus* showing significant quantitative differences when compared to *O. africanus* and *O. grandiflorus*. Figure 7 shows the chromatographic profiles of the species with the marker compounds highlighted.

### 2.4. The GCxGC–ToF–MS and Headspace Analysis

#### 2.4.1. Headspace Profiles of the Volatile Compounds in the Flowers

**Figure 8:**
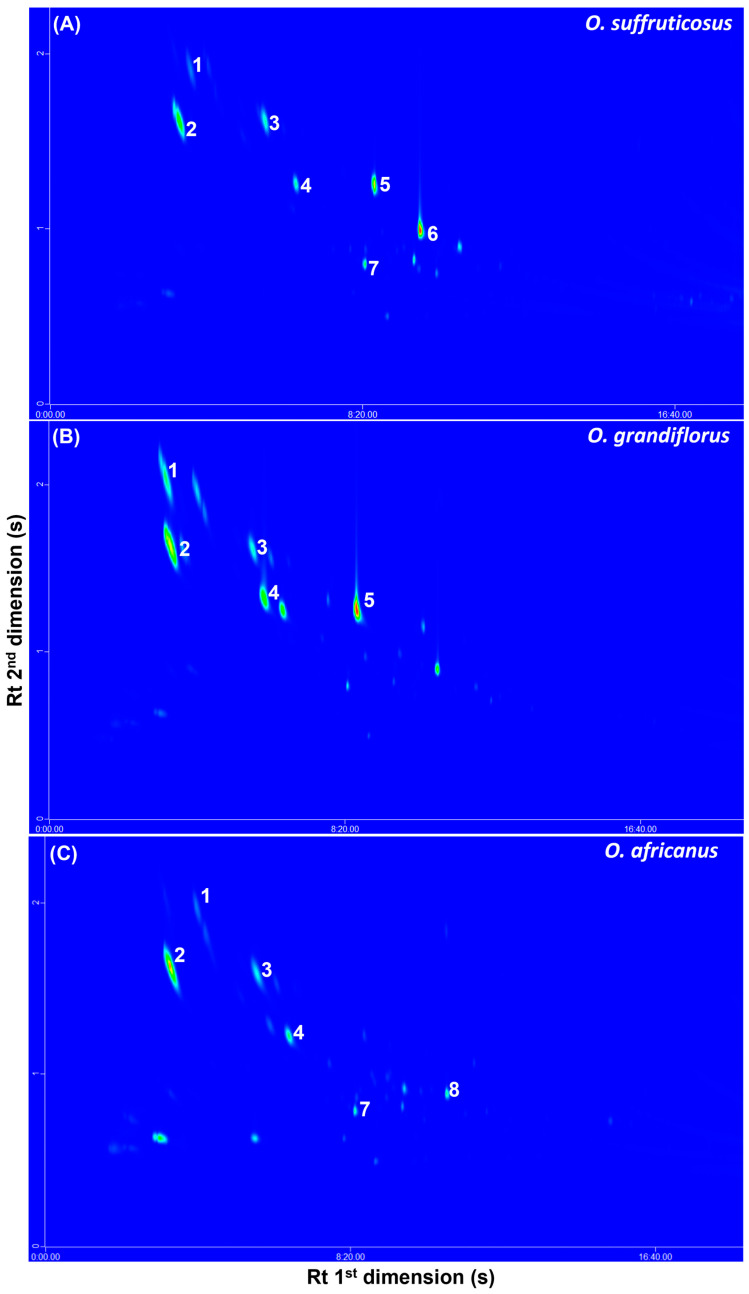
Typical headspace GCxGC-MS contour plots of *O. suffruticosus* (**A**), *O. grandiflorus* (**B**), and *O. africanus* (**C**), indicating the major co-occurring volatile constituents in flowers, namely, (1) α-pinene, (2) α-ocimene, (3) eucalyptol, (4) *o*-cymene, (5) artemisia alcohol, (6) camphor, (7) yomogi alcohol and (8) terpinene-4-ol.

The GCxGC–ToF–MS detected approximately 56 volatile compounds in the three *Oncosiphon* species. The identified compounds belong to various classes, such as esters, aldehydes, aliphatic alcohols, monoterpene alcohols, monoterpene ketones, monoterpenes, and sesquiterpenes. Typical GCxGC–MS contour plots of the flower volatiles of the three species are indicated in Figure 8. Some of the major compounds such as α-pinene (1), α-ocimene (2), eucalyptol (1,8–cineole) (3), *o*-cymene (4), artemisia alcohol (5), and terpinen–4–ol (8) occurred across the three species, although variation in the band intensities could be observed. The percentage peak areas of all the compounds in the flowers (≥0.2%) were determined and are listed in Table 6. The major compounds in the flower volatiles of *O. africanus* were identified as α-ocimene (42.1%) and eucalyptol (10.1%), each representing >10% of the total composition. These compounds were also present in other species and none of the identified compounds were found to be unique to *O. africanus.* The *O. grandiflorus* profile showed major compounds, including 2–methyl–2–octen–4–ol (26.3%), artemisia triene (20.3%), artemisia alcohol (15.3%) and α-ocimene (15.9%), and the marker compounds unique to this species were verbenyl acetate, cis–verbenol, chrysanthenone and α–farnesene. *Oncosiphon suffruticosus* consisted of 2,4,6–trimethyl–1,3,6–heptatriene (25.1%), artemisia triene (14.6%), linalyl *o*–aminobenzoate (13.6%) and artemisia alcohol (11.9%) as the major compounds; however, the unique compounds were identified as methacrolein (2.9%), sabinene (4.7%), santolina triene (3.2%), camphene (5.0%), hexanal (2.7%), 1–octen–3–ol (0.3%), bicyclo [2.2.2] octa–2,5–diene, 1,2,3,6–tetramethyl (7.9%), linalool (4.6%), *trans*–*γ*–caryophyllene (0.9%), lavender lactone (0.4%), *endo*–borneol (0.9%), *p*–cymen–8–ol (0.4%) and 8–hydroxycarvotanacetone (1.4%).

#### 2.4.2. The Headspace Profiles of the Volatile Compounds in Stems and Leaves

**Figure 9:**
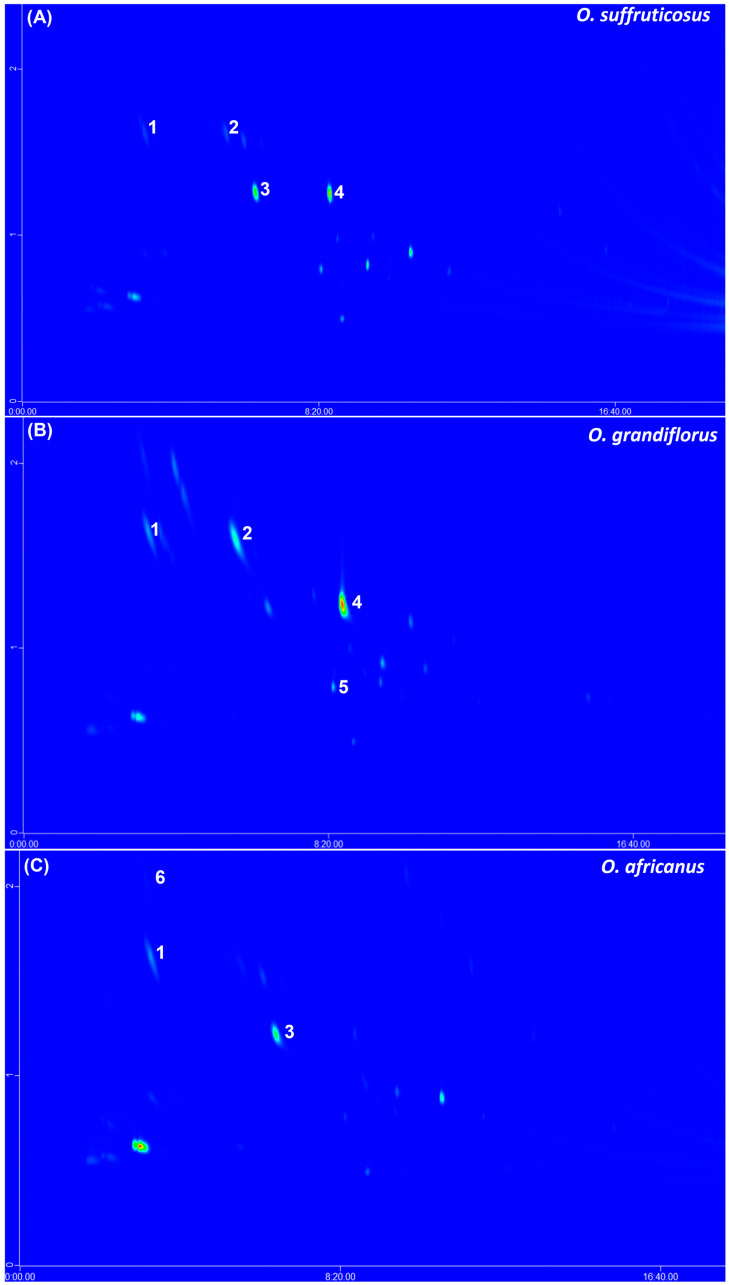
Typical headspace GCxGC-MS contour plots of *O. suffruticosus* (**A**), *O. grandiflorus* (**B**), and *O. africanus* (**C**), indicating major volatile constituents present in stems and leaves, namely, (1) α-ocimene, (2) eucalyptol, (3) *o*-cymene, (4) artemisia alcohol, (5) yomogi alcohol, and (6) α-pinene.

The headspace volatiles of the stems and leaves displayed a similar pattern to that observed for the flowers. The representative contour plots show the major compounds to be α-ocimene (1), eucalyptol (2), *o*-cymene (3), artemisia alcohol (4), and yomogi alcohol (5), which quantitatively varied between the three species (Figure 9). A comparison of the percentage peak areas of all 50 compounds detected in the stems and leaves is provided in Table 7. The major compound constituting at least 10% of the total composition in *O. africanus* was *o*-cymene (10.3%). This is different from the flowers, where α-ocimene (42.1%) and eucalyptol (10.1%) occurred in relatively higher concentrations (Table 6). In *O. grandiflorus*, the main components in the stems and leaves included artemisia triene (20.2%), tetraethylene glycol (17.3%), and α-ocimene (10.1%). Artemisia triene (20.3%) and α-ocimene (15.9%) were also reported as major constituents of the flower volatiles of *O. grandiflorus*, although the flowers were composed of other compounds in higher quantities (Table 6). The stems and leaves of *O. suffruticosus* contained artemisia triene (25.9%), camphor (10.4%), and linalyl o–aminobenzoate (11.8%) as major constituents. Similarly, the flowers exhibited artemisia triene (14.6%) and linalyl o–aminobenzoate (13.6%) as dominant components. In addition, the flowers contained two other prominent compounds, 2,4,6–trimethyl–1,3,6–heptatriene (25.1%) and artemisia alcohol (11.9%), which were present only in trace amounts in the stems and leaves (Table 6).

## 3. Discussion

A few studies have reported on the phytochemical profiles of *Oncosiphon* species, with existing reports focusing primarily on *O. piluliferus* and *O. suffruticosus* [8,9]. The present study provides the first comprehensive phytochemical profiles of the non-volatile and volatile constituents of *O. africanus*, *O. grandiflorus,* and *O. suffruticosus*. The HPTLC flower profiles revealed significant interspecies differences, while intraspecies variation was minimal, suggesting that the chemical composition within each species remains largely conserved. A visual inspection of the HPTLC profiles indicated that these differences were predominantly quantitative rather than qualitative; the species shared similar compounds such as kaempferide but in varying quantities. The HPTLC fingerprinting analysis also provided an initial visual comparison of the non-volatile constituents, indicating that *O. grandiflorus* and *O. africanus* share relatively similar chemical profiles. Among the three species, *O. suffruticosus* exhibited a distinct chemical profile, particularly in the flowers. The presence of more intense bands for phenolic and flavonoid compounds in *O. suffruticosus* suggests that these compounds may be present in higher levels compared to the other two species. Similarly, the HPTLC profiles of stems and leaves revealed quantitative differences in phenolic and terpenoid compounds, with minimal qualitative variation observed within or between species. The use of HPTLC as a complementary tool has proven to be an effective approach for species identification, authentication, and quality control in medicinal plant research. Several studies have successfully used HPTLC as a complementary tool to UPLC–MS to differentiate closely related species. Rubegeta et al. [35] employed HPTLC in combination with UPLC–MS to successfully distinguish *Prunus africanus* samples collected from Cameroon, the Democratic Republic of Congo (DRC), and Zimbabwe.

The LC–MS results revealed that the flowers, stems, and leaves of *O. africanus*, *O. grandiflorus,* and *O. suffruticosus* have several compounds in common; however, these differed quantitatively. In this study, LC–MS served as the primary technique for metabolite annotation and enabled the tentative identification of several classes of non-volatile constituents. Several identified compounds, including amino acids, phenolic acids, flavonoids, sesquiterpene lactones, and fatty acid derivatives, have not been previously documented in the literature, making this study the first to report on the non-volatile compounds present in these three *Oncosiphon* species. Chemometric analysis of the UPLC–MS dataset revealed that the flowers of *O. suffruticosus* exhibited distinct chemical differences compared to *O. grandiflorus* and *O. africanus*. Conversely, some overlap was observed between stems and leaves of *O. suffruticosus* and *O. grandiflorus*, suggesting a degree of chemical similarity in these plant parts. The analysis also identified unique marker compounds responsible for species differentiation, including eupalitin, methyl quercetin, and isorhamnetin-3-*O*-glucoside, which could be used to authenticate *Oncosiphon* species and prevent misidentification. Overall, the variation studies confirmed chemical differences among *O. africanus*, *O. grandiflorus,* and *O. suffruticosus*, indicating that these species cannot be used interchangeably for medicinal applications. The integration of UPLC–MS and chemometric techniques has proven to be a powerful approach for species identification, authentication, and quality control in medicinal plant research.

Headspace volatiles of the three species were found to have several compounds in common, with quantitative differences being more evident. Analysis of the headspace fraction using GC–MS demonstrated that the floral volatiles are dominated by α-pinene, α-ocimene, eucalyptol, o-cymene, and artemisia alcohol, while stems and leaves are primarily characterised by α-ocimene, eucalyptol, and yomogi alcohol. Visual inspection of the 2D–GC contour plots of the species also revealed the presence of quantitative variation within each species. The major compounds (>10%) identified in the plant parts of the three species were mostly terpenes and terpenoids, including *o*-cymene, α-ocimene, artemisia triene, artemisia alcohol, eucalyptol, and camphor. Terpenes and terpenoids are known for their biological activities, such as antimicrobial, anti-inflammatory, anticancer, and analgesic effects [36]. It is interesting to note that the majority of compounds were detected in the headspace of *O*. *suffruticosus* (46), followed by *O. grandiflorus* (32), while the lowest number was detected in *O. africanus* headspace (21). More detailed analysis using GC×GC–MS further showed that *O. suffruticosus* possesses a more complex headspace profile containing additional monoterpenes such as sabinene and camphene. The flowers of *O*. *suffruticosus* also showed additional major constituents that were absent in the stems and leaves.

*Oncosiphon suffruticosus* is the only species where the volatile constituents have been previously reported. Adewinogo et al. [9] used GC–MS to investigate the essential oil composition of *O. suffruticosus*, and identified 16 constituents, in which camphor (31.2%), filifolone (14.0%), chrysanthenone (8.7%), eucalyptol (7.9%), and terpinen–4–ol (7.4%) were reported as major compounds. Their results also highlighted that hydrocarbons and oxygenated monoterpenes were major constituents accounting for 84.64% total composition, with oxygenated monoterpenes being the most abundant at 76.49%. The findings of Adewinogo et al. [9] closely align with the present study, which also identified hydrocarbons and oxygenated monoterpenes as the predominant compounds in the volatile profiles of the three *Oncosiphon* species. Additionally, their study reported eucalyptol and camphor as major constituents in *O. suffruticosus*, which is consistent with our results to some extent, where eucalyptol and camphor were also among the key volatiles identified through headspace analysis. Although one cannot directly compare the headspace data and the essential oil profiles, the headspace technique provides a better representation of the volatiles present in a sample.

While the three species exhibited similar volatile profiles in varying concentrations, the profile of *O. suffruticosus* was notably more complex, with 46 compounds identified. This complexity was further confirmed by complementary techniques such as HPTLC and LC–MS, which also highlighted significant quantitative differences among the species, particularly in the flowers of *O. suffruticosus*. Although these analytical techniques target different chemical fractions and rely on distinct separation and detection principles, their combined interpretation provides consistent evidence of species differentiation. Specifically, the HPTLC fingerprinting together with UPLC–MS chemometric analysis supports closer chemical relatedness between *O. grandiflorus* and *O. africanus*, while LC–MS and GC–MS analyses demonstrate that the phytochemical distinction of *O. suffruticosus* is reflected in both non-volatile and volatile metabolite profiles, indicating that chemical variation among these species occurs across multiple phytochemical classes.

## 4. Materials and Methods

### 4.1. Plant Material Collection

The three *Oncosiphon* species (*O. suffruticosus n* = 28, *O. grandiflorus n* = 16, and *O. africanus n* = 4) were collected during October and November in 2020, from various locations in the Western Cape Province of South Africa (Table 8). A plant collection permit was issued by Cape Nature under permit number CN35–28–17308, and taxonomic verification of the plants was done by a botanist (Prof. John Manning, SANBI). The plants were delivered to the Department of Pharmaceutical Sciences at the Tshwane University of Technology (TUT), where voucher specimens and retention samples are kept.

### 4.2. Plant Material Preparation

The aerial parts of the plants were air-dried at room temperature. Stems and leaves (SL) were separated from the flowers (F) and ground separately into fine powders using a household blender. Randomly selected powdered samples (SL and F) were extracted with methanol: chloroform (1:1, *v*/*v*) (Merck (Pty) Ltd., Johannesburg, South Africa). Each sample (1.00 g) was weighed into a conical flask and extracted with a 10.0 mL volume of the solvent. The flasks were sealed with parafilm and sonicated in an ultrasonic bath (LIBM8, Labcon^®^, Krugersdorp, South Africa) for 20 min. The sonicated samples were filtered through Whatman No. 1 filter paper into pre-weighed conical flasks. The remaining residue was re-suspended in fresh solvent, and the extraction procedure was repeated twice. The filtrates from the three extractions were pooled and evaporated to dryness using a rotary evaporator at 40 °C (Büchi^®^ Rotavapor R–215, Flawil, Switzerland). The samples were further dried using a Genevac^®^ centrifugal evaporator (Genevac^®^, Ipswich, United Kingdom) and stored at 4 °C until further analysis.

### 4.3. High-Performance Thin Layer Chromatography Analysis

The crude extracts were dissolved in UHPLC-grade methanol (ROMIL–UpS^TM^, Johannesburg, South Africa) to a concentration of 10 mg/mL and analysed on a semi-automated high-performance thin layer chromatography system (CAMAG, Muttenz, Switzerland). The system comprised an automatic TLC sampler 4 (ATS4) attached to a nitrogen line, an automatic developing chamber (ADC 2), derivatiser, TLC plate heater (III), visualiser, and documentation device (visualiser 2). Each extract (10.0 mg/mL) was applied in 4 μL volume as 6 mm bands onto HPTLC silica gel 60 F_254_ (20 × 10 cm) plates (Merck (Pty) Ltd., Johannesburg, South Africa) using an autosampler. The bands were positioned at a 10 mm distance from the bottom edge of the plate. Two mobile phases were used for optimum separation on two different plates: solvent system I—toluene: ethyl acetate: formic acid (60:40:1, *v*/*v*/*v*) and solvent system II—ethyl acetate: formic acid: water (100:10:10, *v*/*v*/*v*). The plates were then developed separately in a chamber pre-saturated with each mobile phase for 20 min at 23 ± 2 °C and 40% relative humidity, allowing the solvent to migrate up to 80 mm. The plates were air-dried for 5 min, and then evenly sprayed with derivatising reagents. The anisaldehyde reagent was sprayed on the plate developed using solvent system I, and the natural product (NP) reagent was sprayed on the plate developed using solvent system II. Anisaldehyde reagent was prepared by mixing 85 mL methanol, 10 mL acetic acid, 5 mL sulfuric acid, and 0.5 mL *p*–anisaldehyde (Merck (Pty) Ltd., Johannesburg, South Africa), and the NP reagent was prepared by dissolving 1 g of diphenylboric acid–β–ethylamino ester (Sigma– Aldrich^®^, Johannesburg, South Africa) in 200 mL of ethyl acetate (Merck (Pty) Ltd., Johannesburg, South Africa), and mixing in equal parts with a polyethylene glycol solution prepared by dissolving 1 g of polyethylene glycol–6000 (Sigma–Aldrich^®^, Johannesburg, South Africa) in 200 mL of dichloromethane. VisionCATS 2.5 software was used to operate the instrument, visualise the data, and capture the images [37].

To identify the bands, a separate set of HPTLC plates was developed and visualised. Omitting the derivatisation step, the most intense bands were marked with circles using a pencil, and bands of the same Rf values were scraped off the silica gel plates using a spatula, pooled into a test tube, and dissolved in UHPLC-grade methanol. The mixtures were filtered through a 0.2 μm syringe filter and injected into UHPLC–MS for analysis as described in Section 4.5. Tentative identification of the compounds was carried out using mass spectral fragmentation patterns, molecular formula based on accurate mass, and the PubChem database (https://pubchem.ncbi.nlm.nih.gov, accessed on 22 January 2025).

### 4.4. Liquid Chromatography Quadrupole Time-of-Flight Mass Spectrometry (LC–QToF–MS) for Tentative Identification

The crude extracts (20 mg) were dissolved in 1.0 mL of methanol (Millipore, Bedford, MA, USA), sonicated for 10 min, and then centrifuged for 15 min at 5000 rpm. The supernatant was collected, filtered through a 0.22 µm syringe filter, and then injected (2 µL) into an Agilent liquid chromatographic system (Agilent 1290 series) coupled to 6530 Agilent Accurate–Mass Q–TOF LC/MS with an electrospray ionisation (ESI) interface. The chromatographic separation was achieved on an Agilent SB C8 column (2.1 × 100 mm, 1.8 µm) maintained at 40 °C. The mobile phase consisted of water with 0.1% formic acid (solvent A) and acetonitrile with 0.1% formic acid (solvent B), at a flow rate of 0.23 mL/min. The gradient elution of 10% B to 45% B for 15 min, and in the next 5 min to 100% B was used. The mass spectrometry analysis was performed using the following parameters: drying gas (N_2_) flow rate at 11.0 L/min, temperature at 325 °C, nebulizer at 30 psig, sheath gas temperature at 300 °C, sheath gas flow at 11 L/min, capillary voltage of 3500 V, skimmer at 65 V, Oct RF voltage at 750 V and fragmentor voltage of 125 V. The sample collision energy was set at 40 eV. Agilent MassHunter Acquisition Software controlled the operations, acquisition, and analysis of the data. All data analysis was performed using MassHunter Qualitative Analysis Software Ver. B.07.00 in the mass range of 50–1700 Da. Each sample was analysed in both positive and negative ionisation modes. Accurate mass measurements were obtained using ion correction techniques with reference masses at *m*/*z* 121.0509 (protonated purine) and 922.0098 [protonated hexakis (1H, 1H, 3H–tetrafluoropropoxy) phosphazine or HP–921] in positive ion mode, while *m*/*z* 112.9856 (deprotonated trifluoroacetic acid–TFA) and 1033.9881 (TFA adducted HP–921) were used in the negative ion mode. The compounds were confirmed in each spectrum. For this purpose, the reference solution was introduced into the ESI source via a T–junction using an Agilent Series 1200 isocratic pump (Agilent Technologies, Santa Clara, CA, USA) with a 100:1 splitter set at a flow rate of 20 µL/min. DAD spectra were acquired over a scan range of 190–400 nm.

### 4.5. Ultra-Performance Liquid Chromatography–Mass Spectrometry Analysis for Chemometric Modelling

The crude extracts were dissolved in UPLC-grade methanol (ROMIL–UpS^TM^, Johannesburg, South Africa) to a concentration of 5.0 mg/mL, filtered through 0.22 µm filters, and injected (1.0 μL) into a Waters Acquity I-class UPLC system equipped with a PDA detector (Waters, Milford, MA, USA), in tandem with a mass spectrometer ( Xevo^®^ G_3_ QToF) (Waters, Milford, MA, USA). The chromatographic conditions were optimised to achieve the best peak separation in the shortest run time. Separation was achieved on an Acquity UPLC BEH C18 column (150 mm × 2.1 mm, 1.7 μm particle size; Waters, Milford, MA, USA) maintained at 50 °C. The mobile phase consisted of 0.1% formic acid in water (Solvent A) and 0.1% formic acid in acetonitrile (Solvent B) at a flow rate of 0.3 mL/min. Gradient elution was executed as follows: the initial ratio was 85% A: 15% B, changed to 50% A: 50% B in 12.0 min, then to 5% A: 95% B in 1.0 min, and back to the initial ratio in 0.5 min. The system was equilibrated for 1.5 min, and the total run time was 15 min. The results obtained in negative electrospray ionisation mode were evaluated. Nitrogen was used as the desolvation gas at a flow rate of 600 L/h. The capillary voltage was set to 2450 V, and the sampling cone voltage was 36 V. A desolvation temperature of 450 °C and a source temperature of 100 °C were applied. Spectral scanning was carried out between 100 and 1500 *m*/*z*. The chromatographic software Masslynx v4.2 was used to capture and process all the chromatographic data. Tentative identification of the compounds was carried out using mass spectral fragmentation patterns, molecular formula based on accurate mass, and the PubChem database.

### 4.6. Two-Dimensional Gas Chromatography Coupled to Time-of-Flight and Mass Spectrometry Analysis

A fixed mass (0.50 g) of each powdered sample (SL and F) was weighed into a 20 mL headspace vial (Restek, Bellefonte, PA, USA) and directly analysed in duplicate using GCxGC–ToF–MS system. The analysis was performed on a Leco Pegasus 4D multidimensional GC system (Leco Africa (Pty) Ltd., Kempton Park, South Africa), equipped with a multi-purpose sampler (Gerstel, Mülheim an der Ruhr, Germany), which was operated in the headspace mode. The system comprised an Agilent 7890 gas chromatograph with a cryogenic thermal modulator and a secondary oven. A 30 m × 0.25 mm × 0.25 µm film thickness Stabilwax^®^ polyethylene (Restek, Bellefonte, PA, USA) column served as the primary column, while the secondary column was a 0.790 m × 0.25 mm × 0.25 µm film thickness, Rxi^®^–5Sil MS capillary column (Restek, Bellefonte, PA, USA). The GCxGC–ToF–MS system was equipped with a 1000 µL gas syringe and a sampling tray with a 32-headspace vial capacity (20 mL; Restek, Bellefonte, PA, USA). After placing the powders in the vials, heat was applied at 50 °C with agitation for 15 min in a pre-heating module. A 1000 μL headspace volume was then collected from each vial and introduced into the GCxGC system, applying a 5:1 split ratio, with a front inlet septum purge flow of 3.0 mL/min, and a purge valve time of 60 s following the start of the run. The inlet temperature was maintained at 200 °C. Helium was used as the carrier gas at a constant flow rate of 1.5 mL/min. The primary column was programmed with an initial oven temperature of 40 °C held for 1 min, which was increased at 10 °C/min to 220 °C and held for 2 min. The secondary column temperature was initially held at 60 °C for 0.50 min, and then ramped at 10 °C/min to 240 °C and held for 2 min. The initial temperature of the thermal modulator was set at 80 °C for 1 min, then increased at 10 °C/min to 260 °C and held for 2 min. The total analysis time was 25 min. Both the front MS inlet and transfer line temperatures were held constant at 200 °C and 225 °C, respectively, while the ion source chamber was maintained at 200 °C. The mass spectrometer mass range was 45–550 *m*/*z*, with an acquisition rate of 100 spectra/s. Tentative identification of the compounds was performed by comparing MS spectra with the NIST 11 spectral library. Library similarity factors were determined for both forward and reverse searches. A match of ≥800 was required before the identification was assigned. A minimum signal-to-noise ratio (S/N) cut off >100 was used for data processing.

### 4.7. Chemometrics Data Analysis

The UPLC–MS data were pre-processed in MS–DIAL v4.9 software, which enables the analysis of the entire chromatographic peaks and mass spectra. The aligned data were then exported to SIMCA^®^–P+ 14.0 (Umetrics AB, Umeå, Sweden) software for chemometrics analysis. Principal component analysis (PCA) was applied to the pre-processed data to investigate possible clusters, groups, and trends as determined by the chemical composition of the samples in the principal component space (Granato et al. [38]). The PCA was constructed using the Pareto-scaling method, and a scores plot was evaluated for chemical relationships between the samples. Following PCA, orthogonal projections to latent structures–discriminant analysis (OPLS–DA) was applied to assess chemical variation between the three species, and to identify the chemical information related to these differences [11]. The samples were grouped into three classes according to species identity to generate a Y-variable representing the classification categories [39]. The OPLS–DA model was then constructed on Pareto-scaling data, separating the predictive X-data from variation in X that is not related to differentiating the species. The model was evaluated according to the number of principal components (A) (predictive + orthogonal), cumulative X-variation modelled from A components (R^2^X_cum_), cumulative Y-variation modelled from A components (R^2^Y_cum_), and cumulative variation in X or Y that can be predicted by A components. The loadings plot was used to identify marker compounds responsible for the differences between the three species. To validate the predictive ability of the OPLS–DA model, external validation was carried out by predicting the identity of a new set of samples.

## 5. Conclusions

This study presents the first in-depth phytochemical profiling of three medicinally important *Oncosiphon* species, revealing distinct chemical differences in both the volatile and non-volatile constituents across the species and plant parts. Notably, *O. suffruticosus* exhibited the most chemically distinct profile, particularly the flowers, indicating its potential for greater medicinal value. These findings support the importance of species-specific phytochemical profiling for accurate identification, authentication, and quality control in medicinal plant research.

## Figures and Tables

**Table 1:** The HPTLC bands and compounds identified in *Oncosiphon* flowers.

Rf Value	Band Colour	[M-H]^−^ *m*/*z*	Identification	Occurrence
Anisaldehyde (under white light)				
0.16	Light purple	359.0775	5,6,4′–trihydroxy–7,8,3′–trimethoxyflavone	*O. africanus* *O. grandiflorus*
0.20	Light purple	315.0506	Isorhamnetin isomer	*O. africanus* *O. grandiflorus*
0.28	Yellow	299.0547	Kaempferide	All three species
0.35	Light purple	329.0760	Tricin–glucuronoside	*O. africanus* *O. grandiflorus*
0.42	Light purple	329.0682	Eupalitin	*O. suffruticosus*
0.45	Light purple	–	Unknown	*O. suffruticosus*
0.52	Purple	–	Unknown	*O. africanus* *O. grandiflorus*
0.63	Light purple	–	Unknown	All three species
0.70	Purple	–	Unknown	All three species
0.90	Pink	–	Unknown	All three species
0.95	Pink	–	Unknown	All three species
Natural product reagent (at 366 nm)				
0.08	Yellow	–	Unknown	*O. grandiflorus* *O. suffruticosus*
0.19	Yellow	–	Unknown	*O. suffruticosus*
0.22	Dark blue	–	Unknown	All three species
0.30	Light blue	–	Unknown	All three species
0.32	Yellow	477.1028	Rhamnetin sophoroside	All three species
0.37	Yellow/Green	–	Unknown	All three species
0.42	Dark Yellow	–	Unknown	All three species
0.58	Light blue	–	Unknown	All three species
0.62	Blue	–	Unknown	*O. suffruticosus*
0.68	Blue	–	Unknown	*O. suffruticosus*
0.70	Blue	–	Unknown	All three species
0.72	Blue	515.1194	Dicaffeoylquinic acid	All three species
0.80	Light blue	–	Unknown	*O. africanus*
0.82	Blue	–	Unknown	All three species
0.85	Yellow	315.0506	Isorhamnetin isomer	All three species
		345.0600	Syringetin or spinacetin	All three species
		299.0547	Kaempferide	All three species
		359.0762	5,6,4′–Trihydroxy–7,8,3′–trimethoxyflavone	All three species
0.90	Red	–	Unknown	*O. grandiflorus*
0.97	Red	–	Unknown	All three species

**Table 2:** The HPTLC bands and compounds identified in *Oncosiphon* stems and leaves.

Rf Value	Band Colour	[M-H]^−^ *m*/*z*	Identification	Occurrence
Anisaldehyde (under white light)				
0.12	Light purple	–	Unknown	All three species
0.16	Light purple	359.0775	5,6,4′–trihydroxy–7,8,3′–trimethoxyflavone	*O. africanus* *O. grandiflorus*
0.24	Light purple	–	Unknown	*O. africanus* *O. grandiflorus*
0.28	Yellow	299.0547	Kaempferide	All three species
0.35	Light purple	329.0760	Unknown	*O. grandiflorus* *O. suffruticosus*
0.45	Light purple	–	Unknown	*O. suffruticosus*
0.49	Light purple	–	Unknown	*O. africanus* *O. grandiflorus*
0.52	Light purple	–	Unknown	*O. africanus* *O. grandiflorus*
0.63	Purple	–	Unknown	All three species
0.73	Purple/Pink	–	Unknown	All three species
0.84	Yellow	–	Unknown	*O. grandiflorus*
0.90	Pink	–	Unknown	All three species
0.95	Pink	–	Unknown	All three species
Natural product reagent (at 366 nm)				
0.19	Blue	–	Unknown	All three species
0.28	Light blue/Yellow	–	Unknown	All three species
0.30	Yellow	–	Unknown	All three species
0.37	Light yellow	–	Unknown	*O. africanus* *O. suffruticosus*
0.39	Yellow	–	Unknown	All three species
0.40	Light yellow	–	Unknown	All three species
0.42	Light yellow	–	Unknown	*O. africanus* *O. suffruticosus*
0.48	Light blue	–	Unknown	*O. africanus*
0.53	Light blue	–	Unknown	All three species
0.56	Light blue	–	Unknown	*O. africanus*
0.72	Blue	515.1194	Dicaffeoylquinic acid	All three species
0.81	Light blue	–	Unknown	All three species
0.83	Yellow	313.0712	Dihydroxy–dimethoxyflavone	All three species
		315.0506	Isorhamnetin isomer	All three species
		345.0600	Syringetin or Spinacetin	All three species
		299.0547	Kaempferide	All three species
0.86	Red	–	Unknown	*O. africanus* *O. grandiflorus*
0.90	Light blue/Blue	–	Unknown	All three species
0.95	Red	–	Unknown	All three species

**Table 3:** Putative identification of compounds in leaves (L) and flower (F) extracts of *O. africanus*, *O. grandiflorus*, and *O. suffruticosus* using LC–QToF–MS in positive and negative ionisation modes.

#	RT (min)	Compound Name	Molecular Formula	Mass	[M+H]^+^	Fragment Ions (Positive Mode)	[M-H]^−^	Fragment Ions (Negative Mode)	Source
Amino acids									
**1**	1.17	Serine ^a^ [17]	C_3_H_7_NO_3_	105.0426	106.0501 (106.0499) *	88.0392 [M+H-H_2_O]^+^, 70.0289 [M+H-2H_2_O]^+^	–	–	OAF, OAL, OGF, OGL, OSF, OSL
**2**	1.19	Proline ^a^ [17]	C_5_H_9_NO_2_	115.0633	116.0701 (116.0706)	70.0659 [M+H-H_2_O-CO]^+^	–	–	OAF, OAL, OGF, OGL, OSF, OSL
**3**	1.21	Valine ^a^ [17]	C_5_H_11_NO_2_	117.079	118.0857 (118.0863)	72.0818 [M+H-H_2_O-CO]^+^, 55.0559 [M+H-H_2_O-CO-NH_3_]^+^	–	–	OAF, OAL, OGF, OGL, OSF, OSL
**4**	1.22	Tyrosine ^a^ [17]	C_9_H_11_NO_3_	181.0739	182.0810 (182.0812)	165.0541 [M+H-NH_3_]^+^, 147.0433 [M+H-NH_3_-H_2_O]^+^, 136.0752 [M+H-H_2_O-CO]^+^, 123.0435 [M+H-NH_3_-H_2_O-CH_2_CO]^+^, 119.0493 [M+H-H_2_O-CO-NH_3_]^+^, 95.0491 [M+H-NH_3_-H_2_O-CH_2_CO-CO]^+^	–	–	OAF, OAL, OGF, OGL, OSF, OSL
**5**	1.22	Isoleucine/leucine ^a^ [17]	C_6_H_13_NO_2_	131.0946	132.1023 (132.1019)	86.0961 [M+H-H_2_O-CO]^+^, 69.0688 [M+H-H_2_O-CO-NH_3_]^+^	–	–	OAF, OAL, OGF, OGL, OSF, OSL
**6**	1.66								
**7**	1.31	Threonine ^a^ [17]	C_4_H_9_NO_3_	119.0582	120.0659 (120.0655)	102.0541 [M+H-H_2_O]^+^, 74.0603 [M+H-H_2_O-CO]^+^	–	–	OAF, OAL, OGF, OGL, OSF, OSL
**8**	1.45	Pipecolic acid ^a^ [18]	C_6_H_11_NO_2_	129.0790	130.0869 (130.0863)	112.0761 [M+H-H_2_O]^+^, 84.0814 (C_4_H_6_NO) [M+H-H_2_O-CO]^+^, 56.0497 [C_2_H_2_NO+H]^+^	–	–	OAF, OAL, OGF, OGL, OSF, OSL
**9**	1.96	Phenylalanine ^a^ [17]	C_9_H_11_NO_2_	165.0790	166.0865 (166.0863)	120.0824 [M+H-H_2_O-CO]^+^, 103.0548 [M+H-H_2_O-CO-NH_3_]^+^, 93.0691, 91.0547	–	–	OAF, OAL, OGF, OGL, OSF, OSL
**10**	2.87	Tryptophan ^a^ [17]	C_11_H_12_N_2_O_2_	204.0899	205.0971 (205.0972)	188.0702 [M+H-NH_3_]^+^, 170.0622 [M+H-NH_3_-H_2_O]^+^, 146.0589 [M+H-CH_2_CO]^+^, 143.0728 [M+H-NH_3_-H_2_O-CO_2_]^+^, 118.0638 [M+H-CH_2_CO-CO]^+^, 91.0527 [M+H-CH_2_CO-CO-HCN]^+^	–	–	OAF, OAL, OGF, OGL, OSF, OSL
Phenolic acids									
**11**	1.15	Quinic acid [19]	C_7_H_12_O_6_	192.0634	-	–	191.0559 (191.0561)	173.0459 [M-H-H_2_O]^−^, 129.0571 [M-H-H_2_O-CO_2_]^−^, 111.0468 [M-H-2H_2_O-CO_2_]^−^, 93.0352, 87.0094	OAF, OAL, OGF, OGL, OSF, OSL
**12**	2.50	Neochlorogenic acid/ Chlorogenic acid ^a^/ Cryptochlorogenic acid [20,21]	C_16_H_18_O_9_	354.0951	355.1027 (355.1024) 377.0844 (377.0843) [M+Na]^+^	163.0385 [caffeoyl-H_2_O+H]^+^, 145.0288 [caffeoyl-2H_2_O+H]^+^, 135.0453 [caffeoyl+H-CO_2_]^+^, 117.0347 [caffeoyl+H-H_2_O-CO_2_]^+^	353.0875 (353.0878)	191.0558 [quinic acid-H]^−^, 179.0348 [caffeic acid-H]^−^, 173.0447 [quinic acid-H-H_2_O]^−^, 161.0246 [caffeic acid-H-H_2_O]^−^, 135.0452 [caffeoyl-H-CO_2_]^−^	OAF, OAL, OGF, OGL, OSF, OSL
**13**	3.58								
**14**	3.80								
**15**	4.74	Caffeic acid ^a^ [19]	C_9_H_8_O_4_	180.0423	181.0491 (181.0495)	163.0386 [M+H-H_2_O]^+^	179.0347 (179.0350)	161.0251 [M-H-H_2_O]^−^, 135.0449 [M-H-CO_2_]^−^, 117.0350 [M-H-H_2_O-CO_2_]^−^, 107.0503 (side chain cleavage)	OAF, OAL, OGF, OGL, OSF, OSL
**16**	5.22	Coumaroylquinic acid [20,21]	C_16_H_18_O_8_	338.1002	–	–	337.0928 (337.0929)	191.0555 [quinic acid-H]^−^, 173.0461 [quinic acid-H-H_2_O]^−^, 163.0412 [coumaric acid-H]^−^, 119.0505 [coumaric acid-H-CO_2_]^−^	OAF, OAL, OGF, OGL, OSF (tr), OSL (tr)
**17**	5.68	Feruloylquinic acid [20,21]	C_17_H_20_O_9_	368.1107	-	–	367.1039 (367.1035)	193.0531 [ferulic acid-H]^−^, 191.0559 [quinic acid-H]^−^, 173.0450 [quinic acid-H-H_2_O]^−^, 149.0581 [ferulic acid-H-CO_2_]^−^, 134.0362 [ferulic acid-H-CO_2_-CH_3_]^−^	OAF (tr), OAL, OGF (tr), OGL, OSF (tr), OSL
**18**	7.42	3,4/3,5/4,5–Di–*O*– caffeoylquinic acid ^a^ [20,21]/ DCQA isomer	C_25_H_24_O_12_	516.1268	517.1340 (517.1341)/ 539.1155 (539.1160) [M+Na]^+^	355.1027 [caffeoylquinic acid+H]^+^, 163.0385 [2caffeoyl+H-H_2_O]^+^, 135.0446 [2caffeoyl+H-CO_2_]^+^, 117.0320 [2caffeoyl+H-H_2_O-CO_2_]^+^	515.1193 (515.1195)	353.0876 [caffeoylquinic acid-H]^−^, 335.0764 [caffeoylquinic acid-H-H_2_O]^−^, 191.0559 [quinic acid-H]^−^, 179.0347 [caffeic acid-H]^−^, 173.0452 [quinic acid-H-H_2_O]^−^, 135.0448 [caffeic acid-H-CO_2_]^−^	OAF, OAL, OGF, OGL, OSF, OSL
**19**	7.89								
**20**	8.11								
**21**	8.46								
**22**	9.49	Ferulic acid ^a^ [19]	C_10_H_10_O_4_	194.0579	-	–	193.0510 (193.0506)	178.0261 [M-H-CH_3_]^−^, 149.0611 [M-H-CO_2_]^−^, 134.0379 [M-H-CO_2_-CH_3_]^−^, 117.0345 [M-H-CO_2_-CH_2_-H_2_O]^−^	OAL, OGL, OSL
Flavonoids									
**23**	3.80	Gallocatechin [22]/ Epigallocatechin type flavan-3-ol compounds	C_15_H_14_O_7_	306.0740	–	–	305.0670 (305.0667)	221.0462, 179.0371, 151.0037 (RDA), 137.0249, 125.0221, 123.0099, 109.0291	OAF, OAL, OGF, OGL, OSF, OSL
**24**	3.94								
**25**	5.73	Quercetin–3–*O*–rutinoside (rutin) ^a^, [20]/Quercetin–rhamnosyl–hexoside [20]	C_27_H_30_O_16_	610.1534	611.1604 (611.1607)	465.1025 [M+H-Rha]^+^, 449.1076 [M+H-Glc]^+^, 303.0499 [quercetin+H]^+^, 151.0027, 178.9984	609.1459 (609.1461)	301.0345 (aglycone) [M-H-Rha-Glc]^−^	OAF, OAL, OGL, OSL
**26**	6.30								OAF, OAL, OGF, OGL, OSF, OSL
**27**	5.86	Quercetin–3-*O*-galactoside (Hyperoside)/Isoquercetin/Quercetin–hexoside [20]	C_21_H_20_O_12_	464.0955	465.1025 (465.1028)	303.0505 (aglycone)	463.0881 (463.0882)	301.0351, 300.0270 (aglycone) [M-H-Glc]^−^	OAF, OAL, OGL, OSL
**28**	6.49								OAF, OAL, OGL, OSF, OSL
**29**	6.76								OAF, OAL, OGF, OGL, OSF, OSL
**30**	6.15	Isorhamnetin–dihexoside isomers [21]	C_28_H_32_O_17_	640.1639	641.1709 (641.1712)	479.1198 [M+H-Glc]^+^, 317.0651 [M+H-2Glc]^+^	639.1563 (639.1567)	477.105035 [M-H-Glc]^−^, 315.0507 [M-H-2Glc]^−^,300.0271 [M-H-2Glc-CH_3_]^−^	OAF, OAL (tr) OGF, OGL (tr) OSF, OSL
**31**	6.40								OAF (tr), OAL, OGL, OSF, OSL
**32**	6.72	Luteolin–*O*–rutinoside/Kaempferol–*O*–rutinoside [21]	C_27_H_30_O_15_	594.1585	595.1660 (595.1657)	449.1083 [M+H-Rha]^+^, 287.0555 [M+H-Rha-Glc]^+^	593.1509 (593.1512)	447.0941 [M-H-Rha]^−^, 285.0395 [M-H-Rha-Glc]^−^, 151.0042, 133.0288	OAL (tr), OSF, OSL
**33**	7.25								OSF, OSL (tr)
**34**	7.00	Taxifolin (Dihydroquercetin) ^a^ [23]	C_15_H_12_O_7_	304.0583	305.0651 (305.0656)	–	303.0513 (303.0510)	285.0402 [M-H-H_2_O]^−^, 175.0390, 151.0026, 153.0186, 125.0246, 109.0290	OAF, OAL (tr), OGF, OGL (tr), OSF, OSL
**35**	7.01	Isorhamnetin-rhamnosyl galactoside [20]	C_28_H_32_O_16_	624.1690	625.1760 (625.1763)	317.0655 (aglycone), 153.0151 (RDA A-ring fragment)	623.1619 (623.1618)	477.1048 [M-H-Rha]^−^, 315.0506 [M-H-Rha-Glc]^−^ (aglycone), 300.0268 [M-H-Rha-Glc-CH_3_]^−^, 287.0550 [M-H-Rha-Glc-CO]^−^, 271.0257 [aglycone-H-CO_2_]^−^, 151.0038 (RDA), 107.0130	OAF, OAL, OGF (tr), OSF, OSL
**36**	7.37	Isorhamnetin 3–*O*-rutinoside (Narcissin) ^a^ [20]							OAF (tr), OGF, OGL, OSF, OSL
**37**	7.04	Luteolin (or Kaempferol)–*O*–glucoside/Luteolin–*O*–galactoside [20]	C_21_H_20_O_11_	448.1006	449.1073 (449.1078)	287.0565 (aglycone), 153.0164	447.0930 (447.0933)	285.0401 (aglycone), 257.0428 [M-H-kaempferol-CO]^−^, 229.0470 [M-H-kaempferol-2CO]^−^, 151.0035, 117.0336	OAF, OAL, OGF, OGL, OSF, OSL
**38**	7.72								OAF, OAL (tr)
**39**	7.11	Luteolin–*O*–glucuronide [21,24,25]	C_21_H_18_O_12_	462.0798	463.0869 (463.0871)	287.0543 (aglycone), 153.0175	461.0723 (461.0725)	285.0403 [M-H-GlcA]^−^, 257.0428 [M-H-GlcA-CO]^−^, 229.0450 [M-H-GlcA-2CO]^−^, 151.0050, 117.0339	OAF, OAL, OGF, OGL, OSF, OSL
**40**	7.23	Isorhamnetin 3–*O*–glucoside/Isorhamnetin 3–*O*–galactoside [21]	C_22_H_22_O_12_	478.1111	479.1188 (479.1184)	317.0664 (aglycone), 153.0153	477.1035 (477.1038)	315.0507 (aglycone), 300.0278 [M-H-hexose-CH_3_]^−^, 287.0530 [M-H-hexose-CO]^−^, 151.0040, 107.0145 (RDA fragment)	OAF (tr), OAL, OSF (tr), OSL (tr)
**41**	7.50								OAF, OAL (tr), OGF, OGL, OSF, OSL
**42**	8.69	Isorhamnetin 3-*O*-rhamnoside [26]	C_22_H_22_O_11_	462.1162	463.1231 (463.1235)	317.0661 (aglycone)	461.1091 (461.1089)	315.0517 [M-H-Rha]^−^ (aglycone), 300.0283 [M-H-Rha-CH_3_]^−^, 151.0040 (RDA)	OAF (tr), OAL, OGL, OSL (tr), OSF (tr)
**43**	9.29	Tricin–glucuronoside [25]	C_23_H_22_O_13_	506.1060	507.1137 (507.1133)	331.0812 (aglycone)	505.0985 (505.0988)	329.0666 (aglycone), 314.0426 [M-H-GlcA-CH_3_]^−^, 299.0246, 151.0037 (RDA), 121.0301	OAF, OAL, OSF (tr), OSL
**44**	9.83	Quercetin–feruloylglucoside	C_31_H_28_O_15_	640.1428	641.1497 (641.1501)	–	639.1351 (639.1355)	463.0886 [quercetin glucoside-H]^−^, 300.0267 (aglycone), 301.0358 [M-H-feruloyl-Glc]^−^, 273.0391 [aglycone-H-CO]^−^, 193.0516 [ferulic acid-H]^−^, 151.0037 (RDA)	OAF (tr), OAL, OGL, OSF, OSL
**45**	10.44	Eriodictyol ^a^	C_15_H_12_O_6_	288.0634	289.0710 (289.0707)	271.0605, 153.0179, 135.0422, 119.0432	287.0558 (287.0561)	269.0452 [M-H-H_2_O]^−^, 151.0037 (RDA), 135.0452, 119.0451	OAF (tr), OAL, OGF, OGL, OSF, OSL
**46**	10.74	Quercetin ^a^	C_15_H_10_O_7_	302.0427	303.0500 (303.0499)	285.0369 [M+H-H_2_O]^+^, 275.0540 [M+H-CO]^+^, 257.0443 [M+H-H_2_O-CO]^+^, 149.0233, 153.0181 (RDA)	301.0352 (301.0354)	273.0405 [M-H-CO]^−^, 257.0455 [M-H-CO_2_]^−^, 179.0350, 151.0039, 149.0244, 107.0139	OAF, OAL, OGF, OGL, OSF, OSL
**47**	11.04	Luteolin ^a^	C_15_H_10_O_6_	286.0477	287.0553 (287.0550)	269.0468 [M+H-H_2_O]^+^, 259.0644 [M+H-CO]^+^, 229.0478 [M+H-H_2_O-2CO]^+^, 153.0178 (RDA), 149.0214, 121.0291	285.0401 (285.0405)	257.0432 [M-H-CO]^−^, 241.0500 [M-H-CO_2_]^−^, 227.0327, 151.0035, 149.0233. 133.0290 (RDA)	OAF, OAL, OGF, OGL, OSF, OSL
**48**	12.89	Kaempferol ^a^							OAF, OAL (tr), OSF, OSL (tr)
**49**	11.20	Tetrahydroxy–methoxy flavone (methyl quercetin) [21,22]	C_16_H_12_O_7_	316.0583	317.0661 (317.0656)	302.0399 [M+H-CH_3_]^+^, 274.0483 [M+H-CH_3_-CO]^+^	315.0509 (315.0510)	300.0275 [M-H-CH_3_]^−^, 272.0325 [M-H-CH_3_-CO]^−^, 244.0333 [M-H-CH_3_-2CO]^−^, 179.0349, 151.0036 (RDA)	OAF, OAL, OGF, OGL, OSF, OSL
**50**	11.64								
**51**	13.03	5,7,4′–Trihydroxy flavone (apigenin) ^a^	C_15_H_10_O_5_	270.0528	271.0605 (271.0601)	253.0498 [M+H-H_2_O]^+^, 243.0623 [M+H-CO]^+^, 153.0185, 133.0285 (C_8_H_5_O_2_^+^), 121.0283 (C_7_H_5_O^+^) (RDA)	269.0455 (269.0455)	241.0517 [M-H-CO]^−^, 225.0557 [M-H-CO_2_]^−^, 151.0037 (C_7_H_3_O_4_^−^), 133.0292 (C_8_H_5_O_2_^−^), 117.0343 (C_8_H_6_O^−^)	OAF, OAL, OGL (tr), OSF, OSL
**52**	13.29	Trihydroxy–dimethoxyflavone isomers (Eupalitin) [27]	C_17_H_14_O_7_	330.074	331.0815 (331.0812)	316.0581 [M+H-CH_3_]^+^, 301.0337 [M+H-2CH_3_]^+^, 299.0565 [M+H-CH_3_-OH]^+^, 285.0363 [M+H-CH_3_-H_2_O-CO]^+^, 183.0293, 153.0180 (RDA), 121.0281	329.0666 (329.0667)	314.0420 [M-H-CH_3_]^−^, 299.0192 [M-H-2CH_3_]^−^, 286.0426 [M-H-CH_3_-CO]^−^, 271.0240 [M-H-2CH_3_-CO]^−^, 151.0036 (RDA), 149.0239	OAF, OAL, OGF, OGL, OSF, OSL
**53**	13.66								OAF, OAL, OGF, OGL, OSF, OSL
**54**	14.07								OAF, OAL, OSF, OSL(tr)
**55**	13.90	trihydroxy–trimethoxyflavone	C_18_H_16_O_8_	360.0845	361.0919 (361.0918)	346.0681 [M+H-CH_3_]^+^, 331.0506 [M+H-2CH_3_]^+^, 329.0626 [M+H-CH_3_OH]^+^, 153.0175 (RDA)	359.0775 (359.0772)	344.0529 [M-H-CH_3_]^−^, 329.0301 [M-H-2CH_3_]^−^, 314.0065 [M-H-3CH_3_]^−^, 211.0243, 151.0035 (RDA)	OAF, OAL, OGF, OGL (tr), OSF, OSL
**56**	15.51	Dihydroxy–dimethoxyflavone (Pectolinarigenin/ Cirsimaritin) [24]	C_17_H_14_O_6_	314.0790	315.0860 (315.0863)	300.0640 [M+H-CH_3_]^+^, 153.0181 (RDA), 121.0284	313.0715 (313.0718)	298.0470 [M-H-CH_3_]^−^, 283.0254 [M-H-2CH_3_]^−^, 255.0292 [M-H-2CH_3_-CO]^−^, 227.0341 [M-H-2CH_3_-2CO]^−^, 163.0035 [C_8_H_4_O_4_-H]^−^, 151.0036, 133.0296	OAF, OAL, OGF (tr), OGL, OSF, OSL
Sesquiterpene Lactones									
**57**	6.14	Isomers of Tatridin A(Tavulin, Tabulin)/Tanachin/Deacetyl–Cyclopyrethrosin/Isospiciformin/ Sivasinolide (hydroxylated germacranolides) [28,29,30,31]	C_15_H_20_O_4_	264.1362	265.1431 (265.1434) 282.1699 (282.1700) [M+NH_4_]^+^ 287.1253 (287.1254) [M+Na]^+^	247.1332 [M+H-H_2_O]^+^, 229.1225 [M+H-2H_2_O]^+^, 221.1535 [M+H-CO_2_]^+^, 219.1382 [M+H-H_2_O-CO]^+^, 203.1433 [M+H-H_2_O-CO_2_]^+^, 201.1278 [M+H-2H_2_O-CO]^+^, 185.1328 [M+H-2H_2_O-CO_2_]^+^, 183.1145 [M+H-3H_2_O-CO]^+^,177.0915 [M+H-C_4_H_8_O]^+^, 159.0805 [M+H-C_4_H_8_O-H_2_O]^+^, 149.0965 (C_10_H_13_O^+^), 121.1014 (C_9_H_13_^+^), 105.0699 (C_8_H_9_^+^), 93.0700 (C_7_H_9_^+^)	263.1289 (263.1289)	219.1387 [M-H-CO_2_]^−^	OAF, OAL, OGF, OGL
**58**	6.62								OAF, OAL, OGF, OGL
**59**	6.98								OAF, OAL, OGF, OGL
**60**	8.32								OAF (tr), OAL (tr), OGF (tr), OGL (tr)
**61**	10.64								OAF (tr), OAL (tr), OGF (tr), OGL (tr)
**62**	12.51								OAF, OAL, OGL, OGF
**63**	8.90	Isoepoxyestafiatin and its isomer [28,29,30,31]	C_15_H_18_O_4_	262.1205	263.1280 (263.1278) 285.1101 (285.1097) [M+Na]^+^	245.1179 [M+H-H_2_O]^+^, 219.1388 [M+H-CO_2_]^+^, 201.1272 [M+H-H_2_O-CO_2_]^+^, 173.0964 [M+H-H_2_O-CO_2_-CO]^+^, 145.1016 (C_11_H_13_^+^)	-	-	OAF, OAL, OGF, OGL, OSF (tr), OSL (tr)
**64**	9.10								OSF(tr), OSL
**65**	10.14	4,5-Epoxy-8,13-dihydroxy-1(10),7(11)-germacradien-12,6-olide [28,29,30,31]	C_15_H_20_O_5_	280.1311	281.1379 (281.1384)	263.1277 [M+H-H_2_O]^+^, 245.1142 [M+H-2H_2_O]^+^, 237.1501 [M+H-CO_2_]^+^, 227.1057 (C_15_H_15_O_2_^+^), 201.1271 [M+H-2H_2_O-CO_2_]^+^, 183.1136 [M+H-3H_2_O-CO_2_]^+^, 149.0944 (C_10_H_13_O^+^), 121.1026 (C_9_H_13_^+^), 93.0710 (C_7_H_9_^+^), 91.0530 (C_7_H_7_^+^)	-	-	OGF, OGL
**66**	12.57	Santamarin or its isomer Reynosin (eudesmanolide derivatives) [28,29]	C_15_H_20_O_3_	248.1412	249.1488 (249.1485) 271.1305 (271.1305) [M+Na]^+^	231.1382 [M+H-H_2_O]^+^, 213.1277 [M+H-2H_2_O]^+^, 205.1589 [M+H-CO_2_]^+^, 187.1484 [M+H-H_2_O-CO_2_]^+^, 159.1170 [M+H-H_2_O-CO_2_-CO]^+^, 145.1013 (C_11_H_13_^+^)	-	-	OSF, OSL
**67**	14.58								OAF, OAL, OGF, OGL (tr)
**68**	14.04	Dehydrocostunolide [28,29,30]	C_15_H_18_O_2_	230.1307	231.1381 (231.1380)	213.1276 [M+H-H_2_O]^+^, 187.1481 [M+H-CO-H_2_O]^+^, 185.1326 [M+H-H_2_O-CO-H_2_]^+^, 159.1167 (C_12_H_15_^+^), 145.1009 (C_11_H_13_^+^), 105.0696 (C_8_H_9_^+^), 91.0539 (C_7_H_7_^+^)	-	-	OAF (tr), OAL (tr), OGF (tr), OGL (tr)
**69**	14.21								OAF, OAL, OGF, OGL (tr)
**70**	14.14	2-Oxo-1(10),3,11(13)-guaiatrien-12,6-olide/Dehydroleucodin/Lidbeckialactone/Mesatlantin E (guaianolides) [28,29,30,31]	C_15_H_16_O_3_	244.1099	245.1171 (245.1172) 267.0994 (267.0992) [M+Na]^+^	227.1071 [M+H-H_2_O]^+^, 201.1277 [M+H-CO_2_]^+^, 183.1168 [M+H-H_2_O-CO_2_]^+^, 155.0852, 141.0698 (C_11_H_9_^+^)			OSF, OSL, OAL(tr), OAF (tr)
**71**	14.50								OSF, OSL, OAL(tr), OAF (tr)
**72**	14.54	Matricarin [31] (guaianolide)	C_17_H_20_O_5_	304.1311	305.1386 (305.1384)	287.1253 [M+H-H_2_O]^+^, 245.1175 [M+H–Ac]^+^, 227.1072 [M+H-Ac-H_2_O]^+^, 201.1278 [M+H-Ac-CO_2_]^+^, 183.1166 [M+H-Ac-H_2_O-CO_2_]^+^, 173.0958 (C_12_H_13_O^+^), 145.1013 (C_11_H_13_^+^), 119.0855 (C_9_H_11_^+^), 105.0699 (C_8_H_9_^+^), 91.0540 (C_7_H_7_^+^)	-	-	OSF, OSL
**73**	17.19	1α, 6α-Dihydroxy-4E,10(14)-germacradien-12,8α-olide [28,29,30,31]	C_15_H_22_O_4_	266.1518	-	-	265.1441 (265.1445)	247.1344 [M-H-H_2_O]^−^, 237.1496 [M-H-CO]^−^, 221.1547 [M-H-CO_2_]^−^, 203.1434 [M-H-H_2_O-CO_2_]^−^, 115.0399 (C_5_H_7_O_3_^−^)	OAF, OAL, OGF, OGL, OSF, OSL
**74**	18.61	Costunolide [28,30] (germacranolide)	C_15_H_20_O_2_	232.1463	233.1538 (233.1536)	215.1432 [M+H-H_2_O]^+^, 189.1642 [M+H-CO_2_]^+^, 187.1482 [M+H-H_2_O-CO]^+^, 159.1173 (C_12_H_15_^+^), 145.1013 (C_11_H_13_^+^), 131.0856 (C_10_H_11_^+^), 119.0855 (C_9_H_11_^+^), 105.0698 (C_8_H_9_^+^), 91.0540 (C_7_H_7_^+^)	-	-	OSF, OSL, OAF (tr), OAL (tr), OGF
Fatty acid derivatives									
**75**	12.73	Trihydroxy octadecadienoic acid [32] (TriHODE)	C_18_H_32_O_5_	328.2250	351.2138 (351.2142) [M+Na]^+^	-	327.2177 (327.2177)	309.2070 [M-H-H_2_O]^−^, 291.1977 [M-H-2H_2_O]^−^, 273.1839 [M-H-3H_2_O]^−^, 211.1343 (C_12_H_19_O_3_^−^), 171.1024 (C_9_H_15_O_3_^−^)	OAF, OAL, OGF, OGL, OSF, OSL
**76**	13.73	Trihydroxy octadecenoic acid [32](TriHOME)	C_18_H_34_O_5_	330.2406	353.2295 (353.2298) [M+Na]^+^	-	329.2334 (329.2333)	311.2239 [M-H-H_2_O]^−^, 293.2129 [M-H-2H_2_O]^−^, 275.2016 [M-H-3H_2_O]^−^, 229.1442 (C_12_H_21_O_4_^−^), 211.340 (C_13_H_23_O_2_^−^), 171.1026 (C_9_H_15_O_3_^−^)	OAF, OAL, OGF, OGL, OSF, OSL
**77**	19.31	Hydroxy-octradecadienoic acid (HODE) [33]	C_18_H_32_O_3_	296.2351	-	-	295.2278 (295.2279)	277.2171 [M-H-H_2_O]^−^, 251.2361 [M-H-CO_2_]^−^, 233.2275 [M-H-H_2_O-CO_2_]^−^, 195.1389, 171.1034	OAF (tr), OGF (tr), OSF (tr)
**78**	19.49								OAF, OAL, OGF, OGL, OSF, OSL
**79**	19.99								OAF (tr), OAL (tr), OGF (tr), OGL (tr)
**80**	19.61	9-Octadecenedioic acid [34]	C_18_H_32_O_4_	312.2301	-	-	311.2224 (311.2228)	293.2123 [M-H-H_2_O]^−^, 267.2330 [M-H-CO_2_]^−^, 249.2224 [M-H-H_2_O-CO_2_]^−^, 223.2431 [M-H-2CO_2_]^−^, 233.1555 [M-H-H_2_O-C_3_H_8_O]^−^, 155.0714	OAF, OAL (tr), OGF, OGL (tr), OSF, OSL (tr)
**81**	19.63	13/9-oxo-octadecadienoic acid [33] (oxoODE)	C_18_H_30_O_3_	294.2195	295.2267 (295.2268)	277.2187 [M+H-H_2_O]^+^, 249.2220 [M+H-2H_2_O]^+^, 119.0877, 105.0726, 93.0725, 81.0703	293.2122 (293.2122)	275.2016 [M-H-H_2_O]^−^, 249.2199 [M-H-CO_2_]^−^, 205.1594, 167.1063, 113.0952	OAF, OAL, OGF, OGL, OSF, OSL
**82**	19.78								OAF, OAL, OGF, OGL, OSF, OSL
**83**	19.85								OAF (tr), OAL (tr), OGF (tr), OGL (tr), OSF (tr), OSL (tr)

Note: ‘*’ indicates the theoretical value of exact mass; ‘tr’ indicates the presence of compound in trace amounts (CPS intensity < 10^4^); OAF—*O. africanus* Flower; OAL—*O. africanus* Leaf; OGF—*O. grandiflorus* Flower; OGL—*O. grandiflorus* Leaf; OSF—*O. suffruticosus* Flower; OSL—*O. suffruticosus* Leaf; RDA—retro–*Diels Alder* rearrangement. Glc = 162.0528 Da; Rha = C_6_H_10_O_4_ = 146.0579 Da; GlcA = 176.0321 Da; H_2_O = 18.0106 Da; CH_2_ = 14.0157 Da; CO_2_ = 43.9898 Da; CH_3_ = 15.0235 Da; CO = 27.9949 Da; 2CH_3_ = 30.0470 Da; Ac = 60.0211 Da; CH_2_CO = 42.0106 Da; HCN = 27.0109 Da; NH_3_ = 17.0265 Da. ^a^ Identification based on accurate mass, MS/MS fragmentation pattern, and chromatographic comparison with a reference compound. The superscripts provided with the compound names indicate the reference numbers.

**Table 4:** Putative chemical markers responsible for variation observed within the flowers of the three *Oncosiphon* species.

Species	[M-H]^−^ *m*/*z*	Compound Name
*O. africanus* (pink)	329.066	Eupalitin
	359.076	5,6,4′–trihydroxy–7,8,3′–trimethoxyflavone
	313.071	Dihydroxy–dimethoxyflavone
*O. grandiflorus* (blue)	639.155	Rhamnetin sophoroside
	362.196	Unknown
	557.272	Unknown
*O. suffruticosus* (red)	477.106	Isorhamnetin 3–*O*–glucoside
	315.051	Methyl quercetin

**Table 5:** Potential chemical markers responsible for variation existing between the three *Oncosiphon* species in stem and leaf samples.

Species	[M-H]^−^ *m*/*z*	Compound Name
*O. africanus* (pink)	449.109	Unknown
	515.119	Dicaffeoylquinic acid
	491.081	Unknown
	505.100	Tricin–glucuronoside
	329.066	Eupalitin
*O. grandiflorus* (blue)	479.195	Unknown
*O. suffruticosus* (red)	447.091	Kaempferol glucoside
	315.091	Methyl quercetin
	313.071	Dihydroxy–dimethoxyflavone

**Table 6:** Mean percentage composition (≥0.2% ± SD) of the compounds tentatively identified in the flowers of the three *Oncosiphon* species.

		*O. africanus*	*O. grandiflorus*	*O. suffruticosus*
No	Compound	% Area ± SD (*n* = 8)	% Area ± SD (*n* = 32)	% Area ± SD (*n* = 56)
**1**	Ethyl acetate	ND	1.0 ± 0.5	2.7 ± 1.0
**2**	Methyl acetate	1.2 ± 1.0	3.2 ± 0.7	2.1 ± 1.7
**3**	3–Amino–1–propanol	1.0 ± 0.5	0.7 ± 0.1	1.7 ± 1.8
**4**	Methylene chloride	1.5 ± 0.3	1.5 ± 1.0	1.5 ± 0.6
**5**	Methacrolein	ND	ND	2.9 ± 1.1
**6**	2–methylbutanal	ND	1.9 ± 0.4	2.3 ± 0.4
**7**	α-Pinene	4.3 ± 2.3	4.6 ± 3.2	2.6 ± 0.1
**8**	Sabinene	ND	ND	4.7 ± 0.6
**9**	Santolina triene	ND	ND	3.2 ± 0.9
**10**	1,3,5–Cycloheptatriene	0.8 ± 0.3	1.1 ± 0.8	1.2 ± 0.5
**11**	2,4,6–Trimethyl–1,3,6–heptatriene	ND	1.9 ± 0.7	1.8 ± 0.7
**12**	Camphene	ND	ND	5.0 ± 2.1
**13**	Hexanal	ND	ND	2.7 ± 1.4
**14**	1–Pentanol	2.9 ± 1.1	ND	1.0 ± 0.4
**15**	Eucalyptol (1,8–cineole)	**10.1 ± 1.4**	7.4 ± 2.8	9.5 ± 2.5
**16**	ʎ–Terpinene	1.4 ± 0.7	1.7 ± 1.2	1.7 ± 0.6
**17**	*o*-Cymene	5.5 ± 3.9	6.5 ± 6.4	6.9 ± 4.7
**18**	α-Ocimene	**42.1 ± 2.3**	**15.9 ± 2.2**	**12.2 ± 2.5**
**19**	3–Hexen–1–ol, (Z)–	0.6 ± 0.3	0.3 ± 0.1	0.5 ± 0.3
**20**	*trans*–2,7–Dimethyl–4,6–octadien–2–ol	0.7 ± 0.3	ND	1.1 ± 0.5
**21**	Yomogi alcohol	2.9 ± 0.7	1.6 ± 0.6	4.1 ± 1.6
**22**	Artemisia triene	ND	**20.3 ± 2.1**	**14.6 ± 2.3**
**23**	2,4,6–Trimethyl–1,3,6–heptatriene	ND	5.3 ± 2.0	**25.1 ± 2.4**
**24**	Artemisia alcohol	1.3 ± 0.7	**15.3 ± 1.6**	**11.9 ± 2.8**
**25**	*p*–Cymene	ND	0.6 ± 0.3	0.6 ± 0.2
**26**	Acetic acid	3.1 ± 2.1	3.1 ± 2.6	3.1 ± 2.3
**27**	1–Octen–3–ol	ND	ND	0.3 ± 0.1
**28**	4–Methyl–1,6–heptadien–4–ol	ND	0.9 ± 0.4	1.3 ± 1.2
**29**	2,7–Dimethyl–2,6–octadien–4–ol	1.2 ± 0.4	0.8 ± 0.8	4.1 ± 2.8
**30**	1,6–Heptadien–4–ol, 4–methyl–	ND	0.6 ± 0.3	1.5 ± 0.3
**31**	3,5–Heptadien–2–ol, 2,6–dimethyl–	2.6 ± 0.6	1.6 ± 0.3	0.8 ± 0.3
**32**	Camphor	ND	4.8 ± 3.7	7.7 ± 7.0
**33**	Bicyclo [2.2.2] octa–2,5–diene, 1,2,3,6–tetramethyl–	ND	ND	7.9 ± 1.6
**34**	Linalool	ND	ND	4.6± 1.3
**35**	*trans*–γ–Caryophyllene	ND	ND	0.9± 0.9
**36**	Linalyl *o*–aminobenzoate	ND	0.6 ± 0.3	13.6 ± 1.8
**37**	Chrysanthenone	ND	3.6 ± 3.0	ND
**38**	Verbenyl acetate	ND	3.6 ± 3.0	ND
**39**	Terpinen–4–ol	2.8 ± 1.8	3.8 ± 4.4	3.9 ± 3.2
**40**	α–Farnesene	ND	1.2 ± 0.5	ND
**41**	Lavender lactone	ND	ND	0.4 ± 0.2
**42**	*endo*–Borneol	ND	ND	0.9 ± 0.6
**43**	α–Terpineol	ND	0.7 ± 0.3	1.0 ± 0.4
**44**	*cis*–Verbenol	ND	2.5 ± 1.4	ND
**45**	*p*–Cymen–8–ol	ND	ND	0.4 ± 0.1
**46**	8–Hydroxycarvotanacetone	ND	ND	1.4 ± 0.1
**47**	2–Octen–4–ol, 2–methyl–	ND	**26.3 ± 1.3**	ND

ND: Not detected; Major compounds (>10%) are indicated in bold.

**Table 7:** Mean percentage area (≥0.2% ± SD) of the compounds tentatively identified in stems and leaves of the three *Oncosiphon* species.

	Species	*O. africanus*	*O. grandiflorus*	*O. suffruticosus*
No	Compound	% Area ± SD (*n* = 8)	% Area ± SD (*n* = 32)	% Area ± SD (*n* = 56)
**1**	Ethyl acetate	ND	ND	1.8 ± 0.7
**2**	Methyl acetate	2.5 ± 1.4	ND	2.4 ± 1.2
**3**	Dimethyldiazene	ND	2.9 ± 1.2	1.0 ± 0.4
**4**	3–Amino–1–propanol	ND	ND	0.9 ± 0.5
**5**	Methylene chloride	2.6 ± 1.1	2.1 ± 1.3	2.7 ± 1.5
**6**	Methacrolein	ND	ND	1.8 ± 1.1
**7**	Butanal, 2–methyl–	1.7 ± 0.3	1.0 ± 0.1	1.2 ± 0.4
**8**	α-Pinene	1.6 ± 0.1	ND	ND
**9**	Santolina triene	0.6 ± 0.4	ND	3.2 ± 0.8
**10**	1,3,5–Cycloheptatriene	3.2 ± 1.4	2.6 ± 1.4	1.2 ± 0.6
**11**	2,4,6–Trimethyl–1,3,6 heptatriene	ND	2.3 ± 0.8	2.2 ± 0.8
**12**	Camphene	ND	ND	1.9 ± 0.9
**13**	Hexanal	1.2 ± 0.1	ND	1.2 ± 0.3
**14**	1–Pentanol	ND	ND	0.9 ± 0.3
**15**	Eucalyptol (1,8–cineole)	ND	4.3 ± 1.0	6.9 ± 3.6
**16**	Butyl methyl ketone	ND	ND	8.7 ± 0.0
**17**	ʎ–Terpinene	2.4 ± 1.3	2.3 ± 1.1	1.7 ± 1.0
**18**	*o*-Cymene	**10.3** **± 7.0**	8.6 ± 7.5	8.7 ± 6.2
**19**	Terpinolene	ND	ND	1.6 ± 0.4
**20**	α-Ocimene	5.9 ± 3.4	**10.1 ± 1.1**	2.6 ± 1.1
**21**	Artemesia	ND	1.0 ± 1.5	0.7 ± 0.9
**22**	Yomogi alcohol	ND	1.0 ± 0.8	1.1 ± 0.5
**23**	Artemisia triene	ND	**20.2 ± 6.3**	**25.9** **± 5.2**
**24**	Artemisia alcohol	1.9 ± 1.9	5.7 ± 5.0	7.7 ± 11.6
**25**	*p*–cymenene	0.8 ± 0.3	0.8 ± 0.3	0.7 ± 0.4
**26**	Acetic acid	1.8 ± 0.5	1.2 ± 0.5	1.2 ± 3.3
**27**	1–Octen–3–ol	ND	ND	0.2 ± 0.1
**28**	4–Methyl–1,6–heptadien–4–ol	ND	1.0 ± 0.5	2.5 ± 1.3
**29**	2,7–Dimethyl–2,6–octadien–4–ol	0.3 ± 0.1	0.6 ± 0.4	2.8 ± 1.8
**30**	3,5–Heptadien–2–ol, 2,6–dimethyl–	2.2 ± 0.6	0.6 ± 0.3	1.7 ± 1.5
**31**	Camphor	0.8 ± 0.3	4.5 ± 3.3	**10.4 ± 7.5**
**32**	α–Neoclovene	ND	5.3 ± 4.5	2.4 ± 1.0
**33**	Cadinene	ND	ND	2.8 ± 0.7
**34**	α–Gurjunene	2.1 ± 0.0	ND	4.2 ± 3.8
**35**	Linalool	ND	0.8 ± 0.6	2.8 ± 3.7
**36**	Limona ketone	ND	ND	1.2 ± 0.3
**37**	Linalyl o–aminobenzoate	ND	4.1 ± 3.9	**11.8 ± 16.7**
**38**	Chrysanthenone	ND	3.3 ± 2.6	4.7 ± 5.3
**39**	*trans*–γ–Caryophyllene	ND	1.1± 0.2	0.2± 0.1
**40**	Verbenyl acetate	ND	3.3 ± 2.6	4.7 ± 5.3
**41**	Borneol, acetate	ND	ND	1.2 ± 1.1
**42**	*cis*–Verbenol	ND	9.2 ± 3.4	1.3 ± 0.3
**43**	Terpinen–4–ol	4.2 ± 1.8	5.4 ± 7.8	4.2 ± 3.5
**44**	Caryophyllene	ND	2.3 ± 1.2	ND
**45**	α–Farnesene	1.4 ± 0.5	0.7 ± 0.2	ND
**46**	*endo*–Borneol	ND	0.4 ± 0.2	1.6 ± 1.8
**47**	α–Terpineol	0.4 ± 0.1	1.1 ± 1.3	1.0 ± 0.8
**48**	*p*–Cymen–8–ol	ND	ND	0.3 ± 0.2
**49**	8–Hydroxycarvotanacetone	ND	ND	3.0 ± 1.3
**50**	Tetraethylene glycol	8.3 ± 9.0	**17.3 ± 2.1**	ND

ND: Not detected; major compounds (>10%) are indicated in bold.

**Table 8:** Summary of collection sites, GPS coordinates and voucher specimen numbers of the three *Oncosiphon* species.

Species	Voucher Number	Number of Samples	Locality	GPS Coordinates
*Oncosiphon africanus* (P.J. Bergius) Källersjö	3843	1	Bokbaai	33° 33′ 11.4″ S 18° 18′ 41.0″ E
	3844	1	Velddrif	32° 47′ 28.8″ S 18° 10′ 08.2″ E
	3845	1	Laaiplek	32° 46′ 14.5″ S 18° 09′ 41.8″ E
	3847	1	Cloeteskraal	32° 51’ 39.5″ S 18° 12’ 28.7″ E
*Oncosiphon grandiflorus* (Thunb.) Källersjö	3830 (A–C)	3	Ganzekraal turnoff	33° 31’ 03.1″ S 18° 20’ 49.4″ E
	3832 (A–C)	3	Melkbosstrand turnoff from N7	33° 43’ 31.0″ S 18° 32’ 49.6″ E
	3838 (A–E)	5	28 km along Hopefield road from N7	33° 14’ 10.2″ S 18° 33’ 54.1″ E
	3848 (A–B)	2	Berg River Station	32° 54’ 27.7″ S 18° 20’ 02.5″ E
	3484 (A–C)	3	Berg River Station	32° 54’ 27.7″ S 18° 20’ 02.5″ E
	3828 (A–C)	3	Bokbaai	33° 31’ 37.5″ S 18° 20’ 10.7″ E
	3829 (A–C)	3	Bokbaai	33° 34′ 15.2″ S 18° 19′ 27.7″ E
	3831 (A–C)	3	Blaauwberg farm entrance	33° 43′ 31.2″ S 18° 29′ 25.5″ E
*Oncosiphon suffruticosus* (L.) Källersjö	3837 (A–E)	5	10 km along Hopefield road from N7	33° 23′ 40.0″ S 18° 40′ 26.9″ E
	3839 (A–E)	5	26 km from Hopefield from N7	33° 13′ 53.0″ S 18° 33′ 31.0″ E
	3841 (A–C)	3	Langebaanweg	32° 59′ 42.8″ S 18° 10′ 48.1″ E
	3842 (A–C)	3	Hopefield	33° 13′ 23.0″ S 18° 20′ 35.3″ E
	3846 (A–C)	3	Laaiplek	32° 46′ 14.5” S 18° 09′ 41.8” E

## Data Availability

The raw data supporting the conclusions of this article will be made available by the authors on request.

## Abbreviations

The following abbreviations are used in this manuscript:

ESI	Electrospray ionisation
GCxGC–ToF–MS	Two-dimensional gas chromatography coupled to time-of-flight and mass spectrometry
HCA	Hierarchical cluster analysis
HPTLC	High-performance thin layer chromatography
MS	Mass spectrometry
OPLS-DA	Orthogonal projection to latent structures-discriminant analysis
PCA	Principal component analysis
PDA	Photodiode-array detector
QToF	Quadrupole Time-of-Flight
R^2^X_cum_	Cumulative X-variation
R^2^Y_cum_	Cumulative Y-variation
R*f*	Retardation factor
SD	Standard deviation
TUT	Tshwane University of Technology
UPLC	Ultra-performance liquid chromatography
*v*/*v*	Volume per volume
